# TSTScope Unifies Single‐Cell Multi‐Omics to Identify Functional T Cell States Predictive of Immunotherapy Response

**DOI:** 10.1002/advs.76983

**Published:** 2026-08-05

**Authors:** Shiwei Cao, Jinyu Cheng, Feng‐ao Wang, Chenxin Yi, Jiajun Chen, Keyue Wang, Lulu Liu, Junwei Liu, Yixue Li

**Affiliations:** ^1^ School of Life Science and Technology ShanghaiTech University Shanghai China; ^2^ Guangzhou National Laboratory Guangzhou China; ^3^ Multimedia Laboratory (MMLab) The Chinese University of Hong Kong Hong Kong China; ^4^ Key Laboratory of Systems Health Science of Zhejiang Province School of Life Science Hangzhou Institute for Advanced Study University of Chinese Academy of Sciences Hangzhou China; ^5^ School of Intelligent Systems Engineering Sun Yat‐sen University Shenzhen China; ^6^ Department of Medical Oncology The First Affiliated Hospital Zhejiang University School of Medicine Hangzhou China; ^7^ GMU‐GIBH Joint School of Life Sciences The Guangdong‐Hong Kong‐Macau Joint Laboratory For Cell Fate Regulation and Diseases Guangzhou Medical University Guangzhou China; ^8^ School of Life Sciences and Biotechnology Shanghai Jiao Tong University Shanghai China; ^9^ Shanghai Institute of Nutrition and Health Chinese Academy of Sciences Shanghai China

**Keywords:** cd8, biology, blockade, cancer, computational biology, immune checkpoint, immunotherapy, lung cancer, t cell, transcriptome

## Abstract

Immune checkpoint blockade (ICB) can produce durable responses in cancer, but reliable predictors of benefit are still lacking. CD8^+^ tumor‐specific T cells (TSTs) are essential for ICB efficacy, yet it remains unclear which functional states of these cells are associated with therapeutic benefit. To address this, we developed TSTScope, an interpretable deep learning framework that integrates single‐cell transcriptomic and T‐cell receptor sequencing data to generate unified representations of CD8^+^ T‐cell identity. By applying TSTScope to non‐small cell lung cancer (NSCLC) datasets, we characterized the gene programs defining tumor specificity and computationally inferred a population of potential TSTs (pTSTs). Our analyses show that clinical response is associated with the functional state of these cells rather than their abundance alone. We derived the major pathological response (MPR) score, a metric capturing this functional potential. In an independent validation cohort, the MPR score was associated with pathological response and recurrence‐free survival and provided complementary information to selected response‐associated biomarkers. Collectively, TSTScope identifies a distinct functional state of tumor‐specific T cells linked to ICB response, providing an interpretable framework for studying receptor‐linked T‐cell function in immunotherapy cohorts.

## Graphical abstract

TSTScope is an interpretable AI framework that integrates single‐cell transcriptomes with TCR information through curated gene‐program constraints. By linking receptor context to functional T cell states, it reveals response‐associated tumor‐specific T cell programs in lung cancer immunotherapy cohorts and defines an MPR score associated with pathological response and recurrence‐free survival.

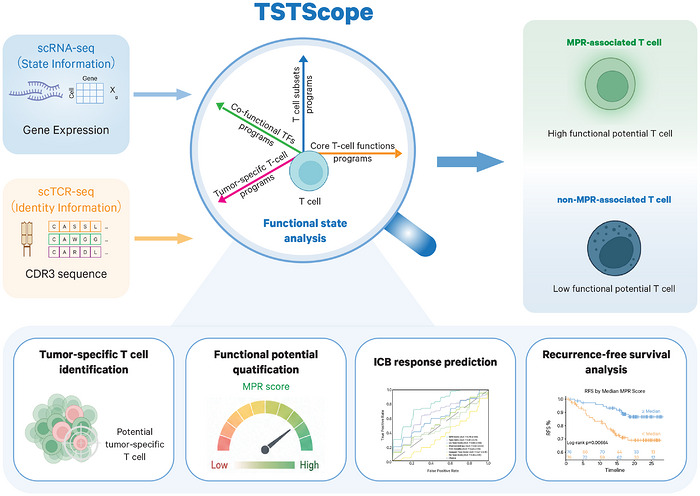

## 1 Introduction

Immune checkpoint blockade (ICB) has reshaped cancer treatment, offering durable responses in a subset of patients. However, the majority fail to benefit, highlighting the critical need for reliable biomarkers to inform treatment decisions [1]. Accumulating evidence positions CD8^+^ tumor‐specific T cells (TSTs) as central mediators of ICB efficacy, given their recognition of neoantigens through T cell receptors (TCRs) [2]. However, the specific cellular states associated with effective anti‐tumor response remain poorly defined.

Recent advances in single‐cell technologies, particularly single‐cell RNA sequencing (scRNA‐seq) and single‐cell T‐cell receptor sequencing (scTCR‐seq), now enable high‐resolution profiling of T‐cell states and clonality. scRNA‐seq captures transcriptional heterogeneity, while scTCR‐seq yields clonal and antigen‐specific insights. In parallel, spatial single‐cell and spatial transcriptomic technologies have begun to resolve tumor‐immune organization, tissue compartments, and cellular neighborhoods in situ. Despite these advances, integrating receptor‐defined tumor reactivity with transcriptional functional state and clinical outcomes remains a major challenge. Spatial transcriptomic datasets provide important tissue‐context information, but standard cohort‐scale datasets generally do not preserve paired TCR CDR3 sequences and therefore cannot directly link clonotype‐defined receptor specificity with cell‐intrinsic functional programs. Existing approaches, including mvTCR [3], scNAT [4], UniTCR [5], and MIST [6], use concatenation or contrastive learning to align multi‐modal data, yet they do not explicitly model the biological coupling between antigen recognition and transcriptional programs. These methods establish the broad paired receptor‐transcriptome integration setting, but they leave open the more specific challenge of linking receptor‐informed tumor‐reactive states to curated functional gene programs and patient‐level response‐associated representations. Furthermore, raw TCR embeddings are often confounded by germline V/J gene usage, masking CDR3‐driven specificity signals [7]. Limited interpretability of existing models further hampers their translation to clinical biomarker development [8, 9].

Consequently, consistent TST identification across heterogeneous datasets remains difficult. Prior strategies have depended on validated gene signatures, including established markers (CXCL13 and ENTPD1/CD39) [10] or predefined TST gene sets, as in Oliveira et al. [11], Lowery et al. (NeoTCR) [12], and Yossef et al. (NeoTCRPBL) [13]. These signatures, however, are sensitive to batch effects and can generalize poorly across studies. Machine learning tools such as MANAscore have also sought to infer potential TSTs (pTSTs) across datasets using known signatures [14]. These approaches remain largely transcriptome‐based, leaving the integration of transcriptional state with TCR‐derived antigen specificity underexplored. Characterizing the functional heterogeneity of TSTs poses a secondary challenge [15, 16]. Although stem‐like precursor exhausted T cells (Tpex) are recognized as pivotal for sustaining ICB responses, a consensus on the precise definition of the Tpex population remains lacking [17, 18]. Current analytical frameworks struggle to resolve TST heterogeneity or elucidate the dynamic trajectories driving the transition from effector states to dysfunction. In particular, quantitative metrics for the “effector potential” of TSTs remain limited, despite their importance for predicting patient outcomes in the context of ICB therapy [19, 20].

To address these limitations, we introduce TSTScope, a multimodal deep learning framework that jointly integrates scRNA‐seq and scTCR‐seq data. TSTScope utilizes a gene program (GP)‐constrained variational autoencoder (VAE) to construct an interpretable latent space that links antigen specificity with transcriptional states. This approach supports TST identification and high‐resolution mapping of their functional heterogeneity. Applying TSTScope to non‐small cell lung cancer (NSCLC) cohorts, we found that the functional context of pTSTs was more closely associated with ICB response than cellular abundance alone. Leveraging these insights, we developed the major pathological response (MPR) score, a metric designed to quantify response‐associated functional potential in pTSTs. The MPR score achieved an area under the curve (AUC) of 0.753 in an independent validation cohort and was associated with recurrence‐free survival. These results provide a quantifiable link between TST heterogeneity and clinical response‐associated states, while motivating further validation across cohorts and cancer types.

## 2 Results

### 2.1 Model Framework of TSTScope

**FIGURE 1:**
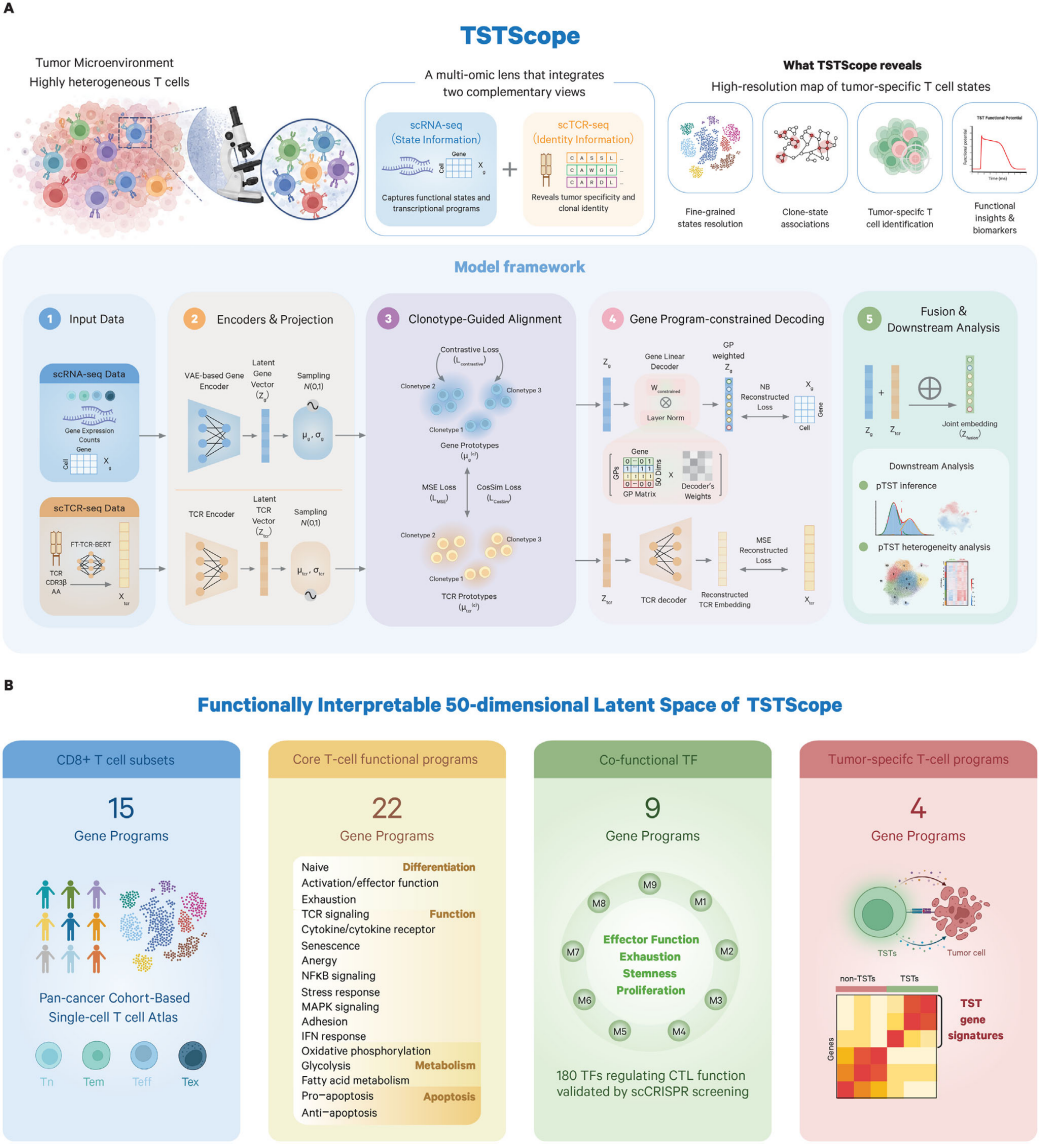
The TSTScope Framework. (A) Step‐wise overview of TSTScope. Paired scRNA‐seq and scTCR‐seq inputs are processed through a GP‐constrained gene‐expression branch and a beta‐chain TCR‐embedding branch. The two modalities are aligned through clonotype‐guided representation learning, reconstructed through gene‐program‐masked and TCR decoders, and integrated into a joint latent representation for TST scoring, pTST calling, and patient‐level response‐state analysis. (B) Functionally interpretable 50‐dimensional latent space of TSTScope organized by four categories of curated T‐cell gene programs.

To address the need for an integrated model combining scRNA‐seq and scTCR‐seq data for functional analysis of TSTs, TSTScope was developed to generate a unified, interpretable representation of T cells by merging transcriptomic profiles with paired TCR repertoires (Figure 1A). Specifically, TSTScope employs a GP‐constrained VAE framework to embed scRNA‐seq data into a 50‐dimensional latent space, where each dimension is explicitly constrained to represent a biologically meaningful T‐cell GP, such as naïve, effector memory, or exhausted states. On the receptor side, TSTScope uses productive TCR β‐chain CDR3 sequences to maximize analyzable cell coverage relative to complete paired α/β cells in public scTCR‐seq cohorts and to capture the V(D)J junctional diversity central to clonotype and antigen‐specificity analysis. These β‐chain CDR3 sequences are embedded using a fine‐tuned TCR‐BERT model [21] and projected into the same latent space through fully connected layers (Methods). To capture the interplay between transcriptional state and TCR specificity, TCR and gene embeddings are aligned through a mixed alignment module prior to integration at clonotype level (Methods). This strategy preserves modality‐specific information while capturing cross‐modal interactions. Latent space interpretability is ensured by the VAE decoder, which reconstructs gene expression solely from the genes within each GP, enforcing each latent variable to correspond to specific biological concept [22]. Additionally, a TCR decoder reconstructs TCR embeddings from the latent space, maintaining fidelity to TCR information, and the joint embeddings from both gene and TCR were used for downstream analysis (Methods).

To enable comprehensive representation of T‐cell functions, particularly for TST cells, the latent space is hierarchically organized around 50 curated T‐cell GPs capturing cellular states from four complementary perspectives (Figure 1B). These include: (i) T‐cell subset programs (15 GPs) representing canonical CD8^+^ T subsets across their life cycle [23]; (ii) Core functional programs (22 GPs) covering essential processes such as TCR signaling, cytokine responses, metabolism, proliferation, and apoptosis [24, 25]; (iii) Co‐functional transcription factor modules (9 GPs) reflecting regulatory circuits of stemness, effector function, proliferation, and exhaustion, derived from large‐scale CRISPR screens [26]; and (iv) Tumor‐specific programs (4 GPs) distinguishing TSTs from bystanders, curated from validated datasets [11, 12, 13, 27] (Table S1). Together, the input modalities, GP‐constrained gene branch, β‐chain TCR branch, clonotype‐guided alignment, and joint latent representation support downstream analysis.

### 2.2 Joint Learning Resolves TCR Antigen Specificity

**FIGURE 2:**
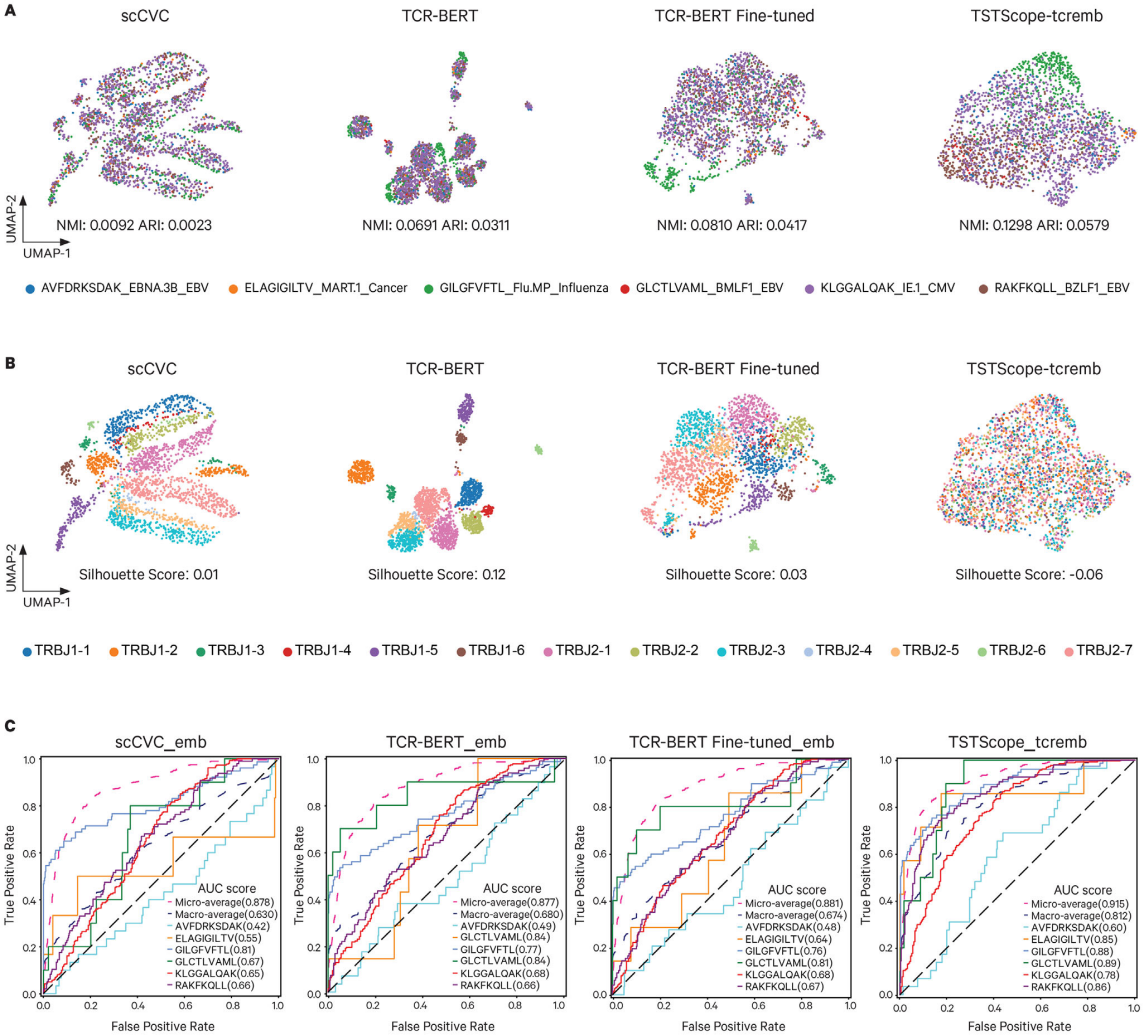
Joint learning improves biological relevance of TCR embeddings. (A) UMAP visualization of TCR embeddings inferred by scCVC, TCR‐BERT, fine‐tuned TCR‐BERT, and TSTScope in the 10x donor antigen‐specificity benchmark. Points are colored by known antigen specificity. Performance is quantified by Normalized Mutual Information (NMI) and Adjusted Rand Index (ARI) scores, as indicated in each panel. (B) UMAP visualization of the same TCR embeddings as in (A), colored by TRBJ gene usage. J‐gene‐associated clustering is quantified by Silhouette score, as indicated in each panel. (C) Receiver operating characteristic (ROC) curves evaluating the ability of different TCR embeddings to predict antigen specificity. Curves show per‐antigen, micro‐average, and macro‐average ROC summaries from the held‐out fold used for visualization.

We evaluated whether TSTScope's joint learning strategy produces biologically meaningful TCR embeddings using a publicly available 10X Genomic dataset with known antigen labels (Methods). We compared the joint learned TSTScope's TCR embeddings with transcriptomic context to its initial embeddings derived from the fine‐tuned TCR‐BERT model [21]. Additionally, we benchmarked these against TCR embeddings inferred from two pre‐trained TCR sequence models, TCR‐BERT [21] and scCVC [7]. Uniform manifold approximation and projection (UMAP) was used to visualize TCR embeddings and related antigen specificities (Figure 2A). To quantitatively assess the intrinsic clustering structure of antigen‐specific T cells, we applied K‐means clustering to the embeddings and calculated the Normalized Mutual Information (NMI) and Adjusted Rand Index (ARI). In this benchmark, TSTScope achieved higher NMI (0.130) and ARI (0.058), indicating improved separation of antigen‐specific populations in the unsupervised space.

Furthermore, embeddings derived solely from TCR sequence were heavily confounded by conserved germline‐encoded J gene segments, leading to clustering driven by germline similarity rather than antigen specificity. In contrast, TSTScope showed reduced J‐gene‐associated clustering, with a Silhouette Score of ‐0.06 (Figure 2B). To further evaluate the embeddings’ discriminative performance, we trained a multi‐class classifier to predict T‐cell antigen specificity under identical stratified five‐fold cross‐validation splits (Methods). The TSTScope TCR‐branch embedding learned with transcriptomic context showed a higher micro‐average AUC (0.915) than the initial fine‐tuned TCR‐BERT embedding (0.881), scCVC (0.878), and TCR‐BERT (0.877) in this benchmark (Figure 2C). Across multiple metrics, including antigen‐specific accuracy, precision, recall, F1 score, and AUC scores, TSTScope showed favorable performance (Figure S1A). These findings support the use of transcriptomic context to reduce germline‐associated artifacts and emphasize CDR3‐related antigen‐specificity signals.

### 2.3 TSTScope Captures Receptor‐Linked Features of Neoantigen‐Specific T Cells

**FIGURE 3:**
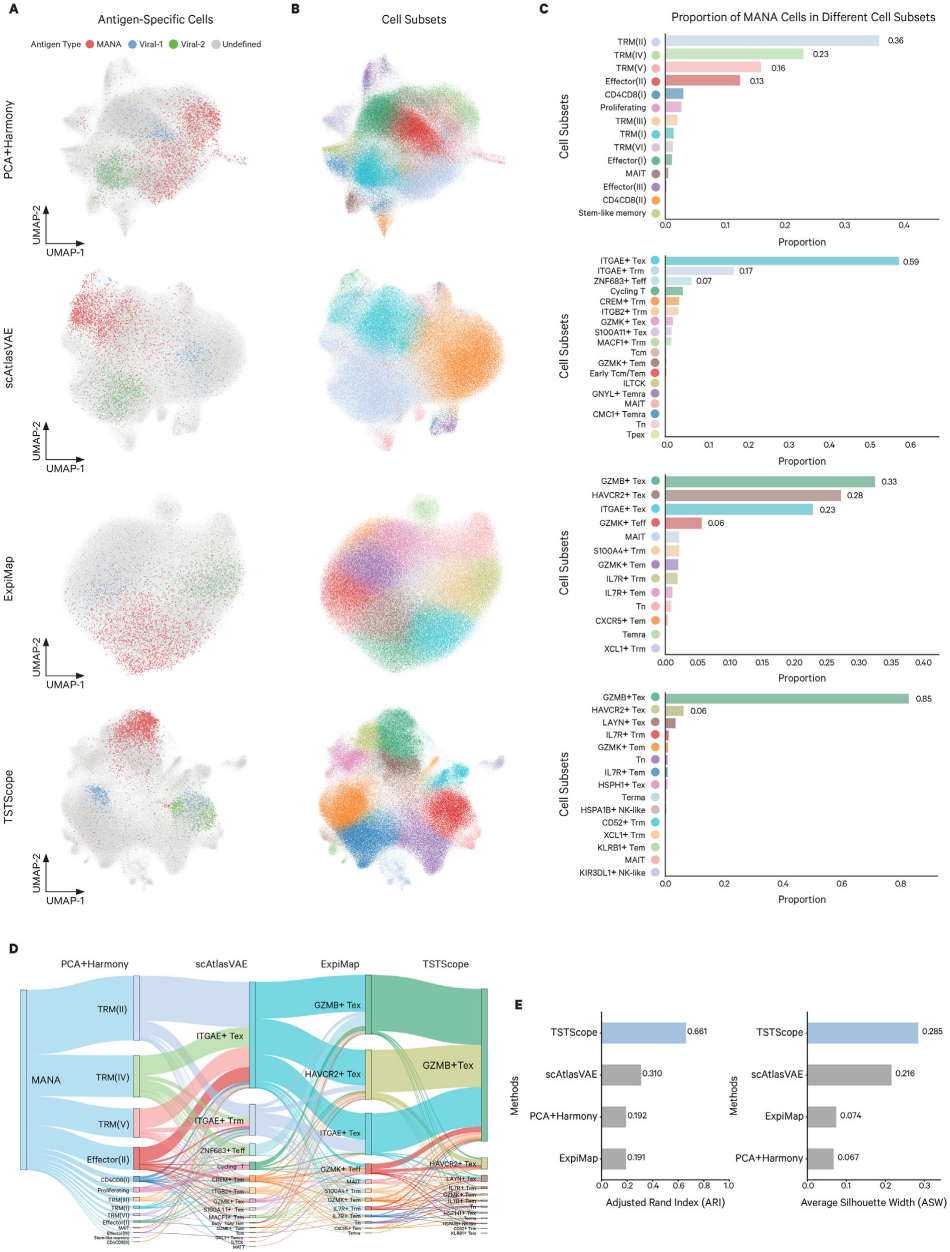
TSTScope enhances the identification and characterization of neoantigen‐specific T cells. (A‐B) UMAP visualizations of 188 804 CD8+ T cells from the Caushi NSCLC cohort (*n* = 16 patients), colored by MANAFEST/ViralFEST antigen labels where available (A) and cell subset annotations (B) across PCA+Harmony, scAtlasVAE, ExpiMap, and TSTScope. (C) Distribution of mutation‐associated neoantigen (MANA)‐specific cells across subsets identified by each method. Numerical labels indicate proportions for subsets exceeding a 5% threshold. (D) Alluvial plot illustrating the mapping and concordance of validated MANA cells from original study clusters (left) through scAtlasVAE and ExpiMap to TSTScope‐defined subsets (right). (E) Quantitative benchmarking of clustering performance across methods using Adjusted Rand Index (ARI) and Average Silhouette Width (ASW), calculated based on ground‐truth antigen specificity labels.

A primary objective of T‐cell repertoire analysis in the Tumor Microenvironment (TME) is to identify T cells that recognize tumor‐specific neoantigens. We next evaluated TSTScope's ability to detect distinct transcriptomic and TCR patterns of TSTs. We utilized a NSCLC dataset from Caushi et al. [28]., comprising 16 patients who underwent paired scRNA‐seq and scTCR‐seq profiling. After quality control, 188 804 CD8^+^ T cells were retained for downstream analysis. Within this cohort, 2083 TSTs and 1420 non‐TST cells from 7 patients were experimentally validated using the mutation‐associated neoantigen functional expansion of specific T cells (MANAFEST) assay and ViralFEST, respectively. We compared TSTScope's joint RNA‐TCR representation with transcriptome‐only baselines, including PCA with Harmony batch correction [29], scAtlasVAE [30], and ExpiMap using the same gene programs as TSTScope [22, 31]. This comparison tested whether adding receptor‐derived specificity and clonotype context improved separation beyond RNA‐defined cell states alone. In UMAP visualizations, TSTScope showed clearer organization of validated antigen‐specific/TST‐like cells than the transcriptome‐only methods (Figure 3A).

We further compared how different approaches captured validated TST enrichment at the cell‐subset level. In the original Caushi et al. annotation, 14 CD8^+^ T‐cell clusters were initially defined, while scAtlasVAE automatically assigned cells to 18 predefined cell subtypes. In contrast, ExpiMap and TSTScope identified 10 and 15 T cell clusters, respectively, with annotations based on prior functional transcriptomic studies of T cells (Figure 3B). Across methods, validated TSTs were enriched in tissue‐resident memory (TRM)‐like subsets with high exhaustion profiles, including TRM(II), TRM(IV), and TRM(V) in the original clustering (Figure 3C and Figure S1B). Similarly, scAtlasVAE assigned approximately 59% and 17% of TSTs to *ITGAE* ^+^ terminally exhausted (Tex) and *ITGAE* ^+^ Trm clusters, respectively. In both ExpiMap and TSTScope, most TSTs were assigned to a *GZMB* ^+^ Tex cluster characterized by cytotoxic potential, exhaustion‐ and tissue‐residency‐related genes, and canonical tumor‐specific T‐cell markers such as *CXCL13* and *ENTPD1* (Figure S2A–G). Notably, TSTScope's joint embedding mapped nearly 85% of validated TSTs to the *GZMB* ^+^ Tex cluster, with an alluvial plot confirming strong concordance with experimentally validated TST labels (Figure 3D). Quantitatively, TSTScope clusters satisfied the dual criteria of high TST purity and broad population coverage, and showed stronger alignment with ground‐truth labels than the compared approaches, with an adjusted Rand index (ARI) of 0.661 and an average silhouette width (ASW) of 0.285 (Figure 3E). These findings support the added value of integrating TCR sequence context with transcriptomic profiles for receptor‐linked TST characterization.

To systematically evaluate the representation‐learning capacity of TSTScope, we used a leave‐one‐patient‐out (LOPO) embedding‐based antigen‐label recovery protocol to test which model constraints supported transferable antigen‐relevant structure (Methods). Using the full Caushi CD8^+^ training cohort and evaluating on the 3503 MANA/Viral antigen‐labeled cells under the matched LOPO evaluation protocol, the TSTScope reference joint embedding achieved LOPO antigen‐label recovery with an AUC of 0.926. Matched component‐removal retraining showed reduced recovery after removing transcriptomic reconstruction (AUC 0.526), clonotype alignment (AUC 0.822), or the gene‐program mask (AUC 0.688) (Figure S3A,B). We next evaluated paired‐chain sensitivity on the 2722 MANA/Viral paired‐chain antigen‐labeled cells (Methods). The subsetted TSTScope reference embedding retained strong recovery (AUC 0.938), close to mvTCR MoE trained with paired‐chain information on 143,698 paired‐chain CD8^+^ cells (AUC 0.956) while retaining TSTScope's gene‐program‐structured interpretability. TSTScope also outperformed the single‐beta‐chain adapted UniTCR joint control (AUC 0.343) (Figure S3C,D).

Because embedding methods can improve apparent biological clustering while retaining patient‐ or tissue‐associated structure, we next evaluated the representation learned in the Caushi CD8^+^ discovery cohort before downstream TST scoring. TSTScope retained measurable patient‐associated local structure, with patient same‐neighbor fraction/iLISI values of 0.494/3.037, compared with 0.228/4.759 for PCA+Harmony, 0.389/3.292 for scAtlasVAE, and 0.272/4.465 for ExpiMap. Tissue‐associated structure was also evident, with tissue same‐neighbor fraction/iLISI values of 0.654/1.706 for TSTScope, 0.561/1.887 for PCA+Harmony, 0.666/1.651 for scAtlasVAE, and 0.568/1.929 for ExpiMap (Figure S3E). Moreover, TSTScope showed the strongest MPR‐associated local preservation, with a same‐label neighbor fraction of 0.716, compared with PCA+Harmony (0.568), scAtlasVAE (0.678), and ExpiMap (0.585) (Figure S3F). Component‐removal patient‐structure diagnostics indicated that these model constraints help limit patient‐associated information in the embedding (Figure S3F). These results indicate that TSTScope preserves antigen‐ and MPR‐relevant local structure while retaining measurable patient‐ and tissue‐associated variation.

### 2.4 Latent Gene Programs Nominate Potential Tumor‐Specific T Cells

**FIGURE 4:**
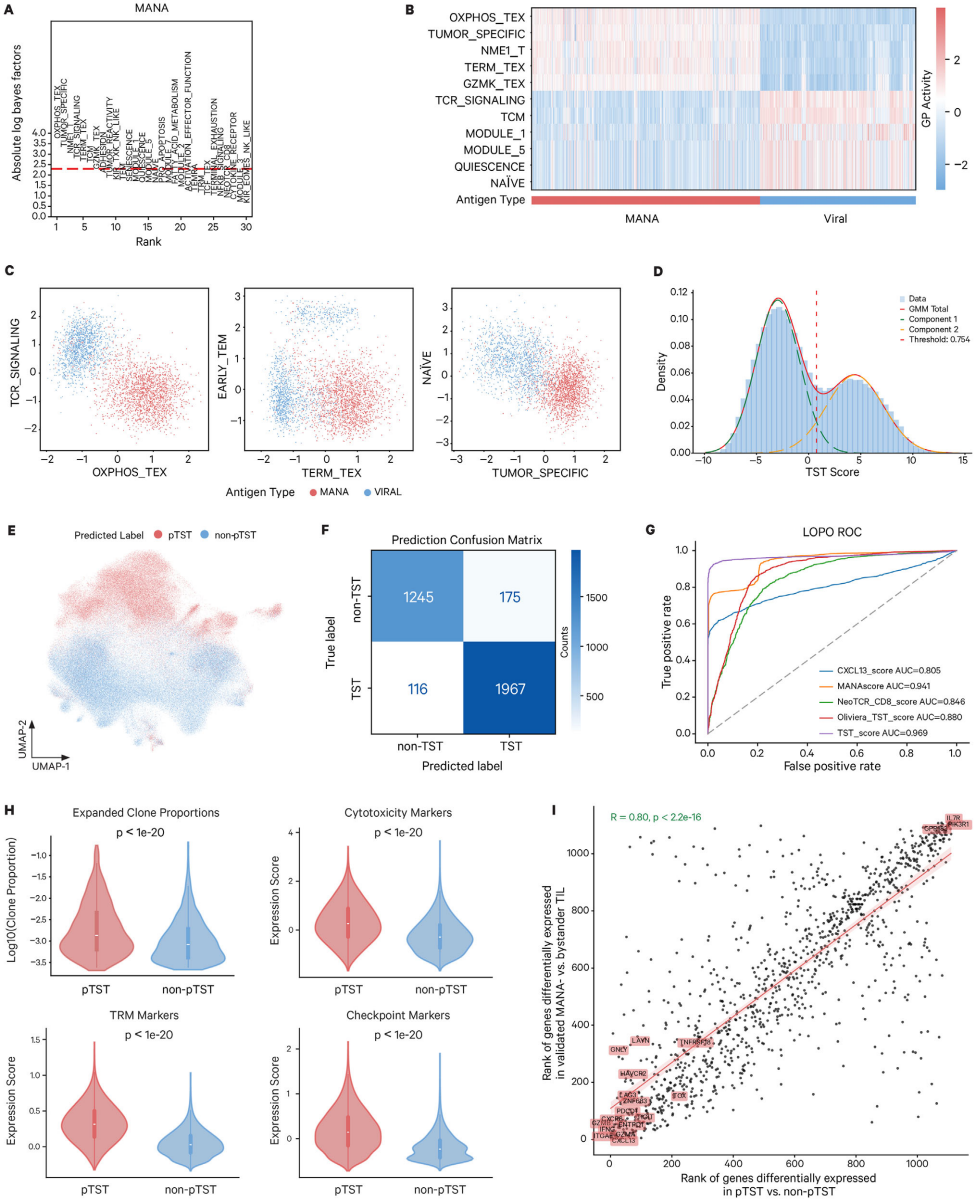
Latent gene programs and TST score accurately predict potential tumor‐specific T cells. (A,B) Latent signature identification showing (A) gene programs (GPs) ranked by absolute log Bayes factor and (B) representative GP activities distinguishing validated MANA‐specific TSTs from viral‐specific non‐TST cells. (C) Bivariate scatter plots of representative GPs illustrating transcriptional separation between tumor‐specific and viral‐specific cell populations. (D) Bimodal distribution of the TST score across the discovery cohort. A two‐component Gaussian Mixture Model (GMM) was used to adaptively define classification thresholds for potential TSTs (pTSTs). (E) UMAP projection of the Caushi NSCLC dataset (*n* = 188,804 cells) colored by GMM‐predicted pTST and non‐pTST labels. (F,G) Performance evaluation of TSTScope using (F) confusion matrix and (G) leave‐one‐patient‐out ROC curve analysis benchmarking the TST score against established biomarkers and TST predictors. AUC values are annotated in the ROC panel. (H) Comparison of predicted pTSTs and non‐pTSTs across clonal expansion, cytotoxicity, tissue‐residency (TRM), and immune‐checkpoint signatures. Statistical significance was assessed by two‐sided Mann‐Whitney U tests with FDR handling for repeated comparison families where applicable. (I) Pearson correlation of differentially expressed gene (DEG) ranks between experimentally validated MANA‐specific cells and TSTScope‐predicted pTSTs. The correlation coefficient and p‐value are labelled.

Leveraging the interpretable, GP‐based latent space of TSTScope, we assessed the distribution of specific GPs to characterize potential TSTs (pTSTs). Enrichment analyses distinguished validated TSTs (MANA‐specific T cells) from non‐TSTs (Viral‐specific T cells). Validated TSTs were preferentially enriched in GPs associated with T‐cell exhaustion and tissue residency (e.g., OXPHOS_TEX, GZMK_TEX, TERM_TEX, ADHESION), as well as tumor‐specific programs (e.g., TUMOR_SPECIFIC, TUMOR_REACTIVITY). Conversely, non‐TSTs showed heightened activity in early memory programs (TCR_SIGNALING, TCM, EARLY_TEM) and stemness related programs (NAÏVE, MODULE_5) (Figures 4A,B). These patterns of gene‐program activity were also consistently preserved within TST‐associated subsets (GZMB+ Tex, HAVCR2+ Tex), and viral‐specific T‐cell‐associated subsets (IL7R+ Trm, GZMK+ Tem) (Figure S4A,B), which align with established characterizations of TST biology [32, 33, 34].

Across validated TSTs and TST‐associated subsets, OXPHOS_TEX, TERM_TEX and TUMOR_SPECIFIC consistently ranked among the most robust positively enriched gene programs, whereas TCR_SIGNALING, EARLY_TEM and NAÏVE represented the most robustly depleted programs. The combined activity of positive and negative gene programs separated validated TSTs from non‐TSTs (Figure 4C and Figure S4C). Additionally, among tumor‐associated gene programs, TUMOR_SPECIFIC showed prominent and consistent enrichment across distinct TST groups, exceeding that of related programs such as TUMOR_REACTIVITY and NEOTCR_CD8 (Figure S4D). This supports TSTScope's ability to resolve distinctions among overlapping gene signatures, thereby highlighting a transcriptional hallmark of TST identity.

Based on these insights, we defined a TST score as the combined activity of positively associated GPs (TUMOR_SPECIFIC, TERM_TEX, OXPHOS_TEX) minus that of negatively associated ones (TCR_SIGNALING, NAÏVE, EARLY_TEM). To identify pTSTs, we employed a dual Gaussian Mixture Model (GMM) to model the bimodal distribution of TST scores, distinguishing high‐scoring pTSTs from the majority of low‐scoring non‐TSTs (Figure 4D). The classification threshold was set at the intersection of the two Gaussian curves. To accommodate sample‐specific variability, thresholds were derived adaptively for each sample, with a global cutoff applied only in the absence of bimodality (Methods). This data‐driven approach reduces reliance on arbitrary thresholds and supports consistent classification across datasets.

In this NSCLC dataset, the GMM‐based classifier identified 74,128 pTSTs with high TST score among 188,804 CD8^+^ T cells. These pTSTs were enriched in subsets containing experimentally validated TSTs (Figure 4E and Figure S4E), and 76.7% originated from tumor tissue, where they were enriched within subsets that also contained experimentally validated TSTs (Figure S4F). Confusion matrix analysis showed concordance with ground‐truth labels, achieving a precision of 93.92% and specificity of 94.30% (Figure 4F). We further performed a LOPO association validation using the stored TST score in antigen‐labeled T cells. In this analysis, the TST score distinguished validated tumor‐specific T cells from viral‐reactive T cells with AUC = 0.969 and AP = 0.985. Comparator transcriptomic scores captured related but lower point‐estimate signal, including MANAscore (AUC = 0.941), Oliveira_TST score (AUC = 0.880), NeoTCR_CD8 score (AUC = 0.846), and CXCL13 score (AUC = 0.805) (Figure 4G and Figure S4G and Table S2). Finally, the inferred pTSTs exhibited hallmark TST features, including enhanced clonal expansion and elevated expression of genes associated with tissue residency (*ITGAE*, *ZNF683*), cytotoxicity (*GZMB*, *GZMA*), and immune checkpoints (*LAG3*, *ENTPD1*, *TIGIT*) compared to non‐pTST cells (Figure 4H), and differential‐expression rank analysis further showed concordance between TSTScope‐inferred pTSTs and experimentally validated TST profiles (Figure 4I).

### 2.5 Functional Potential of pTSTs is Associated With ICB Response

**FIGURE 5:**
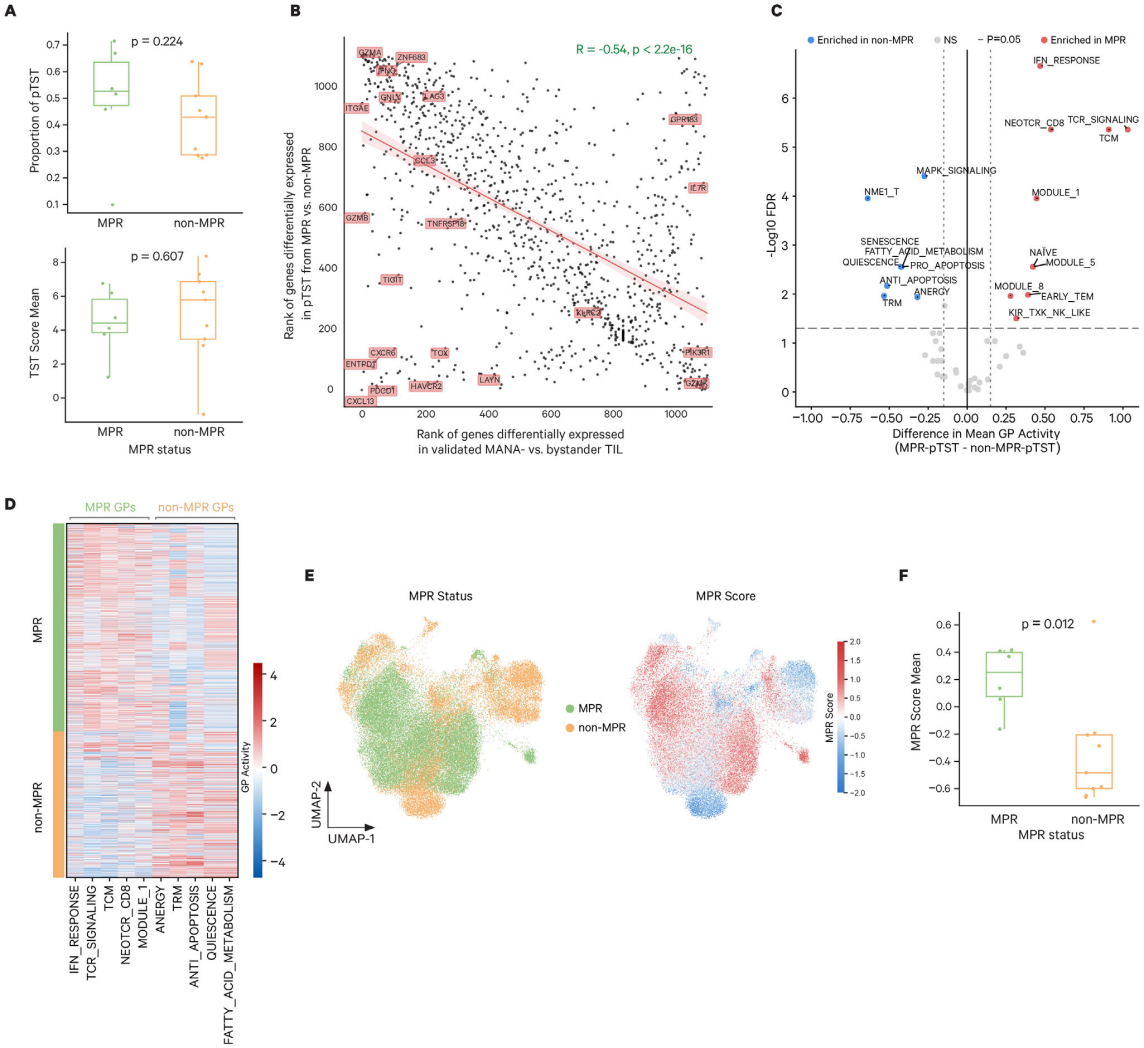
Functional state of pTSTs is associated with clinical response to immune checkpoint blockade. (A) Comparison of the mean proportion of intratumoral pTSTs and mean TST score between patients with major pathological response (MPR; *n* = 6) and non‐MPR (*n* = 9) in the Caushi NSCLC cohort. Statistical significance was assessed by two‐sided Mann‐Whitney U tests. (B) Pearson correlation of DEGs between MPR‐derived vs. non‐MPR pTSTs and validated MANA‐specific vs. bystander TILs. The correlation coefficient and *p* value are indicated. (C) Volcano plot showing differential GP activity from MPR and non‐MPR groups. The horizontal dashed line marks the FDR threshold (*q* < 0.05), and vertical dotted lines mark effect‐size boundaries (|Delta Z| > 0.15) for enriched programs. (D) Heatmap of the top five response‐associated DGPs across pTSTs from MPR and non‐MPR groups. (E) UMAP visualization of intratumoral pTSTs (*n* = 56 885 cells) colored by the clinical response status (left) and the calculated MPR score (right). (F) Comparison of mean intratumoral pTST MPR score between MPR and non‐MPR patients. Statistical significance was assessed by two‐sided Mann‐Whitney U test.

To investigate the relationship between pTSTs and clinical outcomes, we analyzed their abundance and functional states in tumor tissue across ICB response groups in the Caushi et al. dataset. This cohort included tumor samples from 15 patients: 6 with major pathological response (MPR) and 9 with non‐MPR. Neither the proportion of pTSTs in tumor tissue nor their average TST score showed a positive correlation with MPR (Figure 5A). Patients with MPR instead showed a trend toward lower average TST score, suggesting that the abundance of tumor‐reactive T cells alone is insufficient for clinical benefit. A high TST score may capture terminally differentiated states that are refractory to reactivation. This interpretation was further supported by differential gene expression ranking analysis, which showed that pTSTs derived from MPR patients exhibited an overall transcriptional program largely opposite to that observed in validated TSTs (Figure 5B).

We next analyzed the GP profiles of these intratumoral pTSTs to characterize their functional states across different response groups. Differential gene‐program (DGP) analysis revealed marked differences in gene‐program activity between MPR and non‐MPR patients. pTSTs derived from MPR patients were preferentially enriched for gene programs associated with stemness (e.g., NAÏVE, MODULE_1 and MODULE_5) and early activation (e.g., TCR_SIGNALING, TCM and IFN_RESPONSE). By contrast, pTSTs from non‐MPR patients exhibited elevated activity of gene programs typically enriched in TSTs, including those related to terminal exhaustion and dysfunction (e.g., NME1_T, ANERGY, PRO/ANTI_APOPTOSIS and SENESCENCE) (Figure 5C).

Consistently, differences in the activity of the top five DGPs between MPR‐derived pTSTs and non‐MPR‐derived pTSTs separated pTSTs from the two patient groups (Figure 5D). This biological distinction motivated the definition of the “MPR score”, a metric designed to quantify the functional potential of intratumoral pTSTs for evaluating MPR outcome. The MPR score was calculated as the summed activity of top five DGPs identified in pTSTs from MPR patients (IFN_RESPONSE, TCR_SIGNALING, TCM, NEOTCR_CD8, and MODULE_1). Consistent with this definition, pTSTs derived from tumors of MPR patients exhibited higher MPR score (Figure 5E). Moreover, the mean MPR score of intratumoral pTSTs at the individual patient level separated patient groups with distinct responses to ICB therapy (*p* = 0.012) (Figure 5F). These discovery‐cohort results define a receptor‐informed, gene‐program‐based functional‐state score for downstream patient‐level response analysis.

To further elucidate the biological interpretation of the MPR score, we examined its relationship with established stemness‐related and Tpex‐associated gene‐expression signatures (Figure S5A–C). The MPR score was compared with a stemness enrichment score derived from canonical stemness markers (TCF7, IL7R and SELL) [17, 35], as well as three previously reported Tpex gene‐expression scores defined in independent studies [36, 37, 38]. These alternative scores showed less consistent separation of pTSTs derived from MPR versus non‐MPR patients and did not distinguish patient groups at the individual level (Figure S5A,B). Among the Tpex‐related scores, the MPR score showed a positive correlation with the Tpex signature defined by Liu et al., but not with other Tpex scores (Figure S5C). These analyses indicate that the MPR score captures a Tpex‐related but non‐identical response‐associated state.

To test whether this MPR‐score‐defined functional‐state axis was reflected in spatial tumor contexts, we analyzed an independent NSCLC spatial immunotherapy resource from Chen et al. [39]. Chen et al. identified stem‐immunity hubs as response‐associated spatial niches and further used GeoMx Cancer Transcriptome Atlas (CTA) profiling to compare stem‐immunity, tertiary lymphoid structure (TLS)‐like, and immunity‐hub spatial regions of interest (ROIs). This ROI‐level expression resource provided direct coverage for projecting the independently defined MPR score. After within‐case gene standardization, ROI type was strongly associated with the projected MPR score (Kruskal‐Wallis *p* = 5.88e‐09). Stem‐immunity and TLS‐like ROIs showed similarly high values of the independently defined MPR score, and both were higher than immunity‐hub and non‐hub ROIs after FDR correction (Figure S5D). In case‐adjusted logistic regression, each one‐standard‐deviation increase in MPR score was associated with higher odds of stem‐immunity hub location (OR = 1.80, *p* = 5.98e‐05; Figure S5E). This result indicates that our predefined MPR score captured organized immune‐niche activity shared by stem‐immunity and TLS‐like regions. Together, these ROI‐location analyses identify a spatial association between the independently defined MPR score and response‐associated stem‐immunity hub organization and related TLS‐like immune niches.

### 2.6 The MPR Score Captures a Patient‐Level Response‐Associated Functional State

To evaluate whether the MPR‐score‐associated functional state transfers to an independent ICB‐treated NSCLC cohort, we applied the complete TSTScope pipeline, including pTST inference and MPR scoring, to the cohort reported by Liu et al. [40]. (*n* = 234). Patients with fewer than 100 intratumoral CD8^+^ T cells were excluded to support pTST inference. After quality control, 219 188 intratumoral CD8^+^ T cells from 221 patients with paired scRNA‐seq and scTCR‐seq data were retained for analysis (Methods). Among these patients, 220 had available pathological response annotations and were used for patient‐level MPR‐status analyses.

**FIGURE 6:**
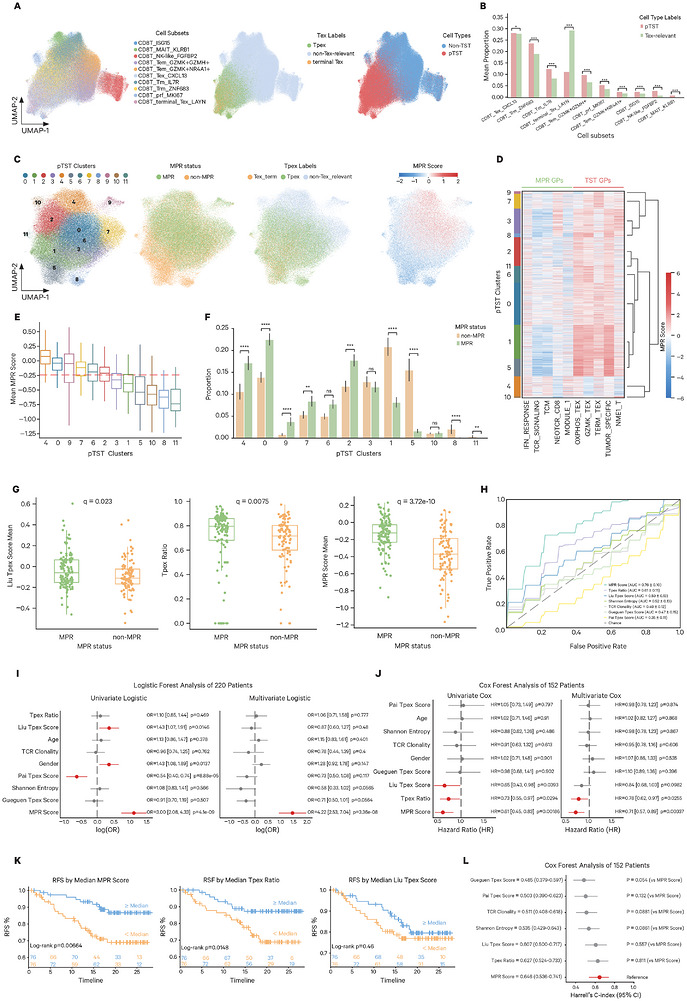
The MPR score captures a patient‐level response‐associated functional state in an independent validation cohort. (A) UMAP projections of CD8^+^ T cells (*n* = 219 188) from the Liu NSCLC cohort, colored by original cell subset annotations (left), study‐defined Tpex/Tex labels (middle), and TSTScope‐inferred pTST status (right). (B) Mean proportions of pTSTs (*n* = 221 patients) and original study‐defined Tex‐relevant populations (*n* = 215 patients) across identified cell subsets. Statistical significance was determined by two‐sided Mann‐Whitney U tests; p‐values are indicated as ^***^ *p* < 0.001 and ^*^ *p* < 0.05. (C) UMAP visualizations of sub‐clustered pTSTs (*n* = 85 400) identified by TSTScope, colored by cell clusters, clinical response status, original Tpex labels, and MPR score. (D) Heatmap plot of relative GP activities across pTST clusters, grouped by MPR‐associated and TST‐associated programs. (E) Boxplot of MPR scores across cells in individual pTST clusters, ordered by mean score magnitude. The red dashed line denotes overall median score across pTST populations. (F) Comparison of per‐patient pTST cluster proportions between MPR (*n* = 123) and non‐MPR (*n* = 97) groups. Statistical significance was assessed by two‐sided Mann‐Whitney U tests with FDR handling for repeated cluster‐composition comparisons where applicable. (G) Patient‐level comparison of Liu Tpex signature score, Tpex ratio, and MPR score between MPR and non‐MPR groups. Benjamini‐Hochberg FDR‐adjusted q values are reported for the displayed group comparisons. (H) ROC curves evaluating the predictive performance of the MPR score vs. Tpex‐ and TCR‐based biomarkers for pathological response. AUC scores are indicated. (I) Forest plot showing score‐only and one‐covariate logistic regression summaries for patient‐level MPR status. Odds ratios (ORs), 95% confidence intervals, and model *p* values are shown. (J) Forest plot showing univariate Cox proportional‐hazards analysis for recurrence‐free survival (RFS) in patients with complete follow‐up annotations (*n* = 152; 30 recurrence events). Hazard ratios (HRs), 95% confidence intervals, and model *p* values are shown. (K) Kaplan‐Meier RFS curves stratified by the median MPR score, median Tpex ratio, or median Liu Tpex score. Log‐rank *p* values are indicated. (L) Cox‐based prognostic comparison of MPR score with Tpex ratio, Liu Tpex score, Pai Tpex score, Gueguen Tpex score, TCR clonality, and Shannon entropy in 152 patients with complete RFS annotations. Harrell's C‐index values with 95% confidence intervals are shown, and *p* values indicate comparison with the MPR score.

Using TSTScope, we identified 85 400 intratumoral CD8^+^ T cells as pTSTs in this cohort (Figure S6A). In the original study, Liu et al. partitioned CD8^+^ T cells into ten subsets and defined Tex‐relevant cells as those sharing TCR clonotypes with terminally exhausted cells (CD8T_terminal_Tex_LAYN). Cells clonally linked to the terminal Tex subset but residing outside this cluster were annotated as Tpex. The pTSTs inferred by TSTScope showed extensive overlap with these Tex‐relevant populations, predominantly mapping to CD8T_Tex_CXCL13 and CD8_Trm_ZNF683 subsets. Notably, pTSTs also included a substantial fraction of non‐Tex relevant cells, mainly originating from CD8T_Trm_ZNF683, CD8T_Tm_IL7R, and CD8T_Tem_GZMK^+^GZMH^+^ cell subsets, with smaller contributions from CD8T_NK‐like_FGFBP2 and CD8T_prf_MKI67 (Figure 6A,B).

To further resolve functional heterogeneity within pTSTs, we partitioned these cells into 12 subsets using the joint latent embedding learned by TSTScope. pTSTs from patients with distinct responses to ICB therapy displayed markedly different cluster preferences. Subsets enriched for non‐Tex‐relevant cells (C4, C7, and C9) were predominantly derived from MPR patients, whereas clusters characterized by terminal Tex features (C1, C5, C8, and C11) were largely contributed by non‐MPR patients. Consistent with these compositional differences, pTSTs from MPR patients exhibited higher MPR scores (Figure 6C and Figure S6B). Moreover, MPR scores exhibited a continuous, graded distribution across pTST subsets rather than discrete stratification. These clusters exhibited hierarchical differences in the activity of MPR‐associated and TST‐associated gene programs: from C4, C0, and C2 to C1 and C5, decreasing MPR‐program activity was accompanied by a progressive activation of TST‐related programs (Figure 6D). Clusters with higher MPR scores were enriched in MPR patients, whereas clusters with lower scores were predominantly derived from non‐MPR patients (Figure 6E,F).

We next benchmarked the MPR score against TCR repertoire metrics, including clonality and Shannon entropy [41, 42], as well as selected established ICB response biomarkers, including three previously reported Tpex signatures and the Tpex ratio defined by Liu et al. In this comparison, Liu's Tpex signature score (q = 0.023), the Tpex ratio (q = 0.0075), and the MPR score (q = 3.72 × 10^−^^10^) were elevated in MPR patients after FDR correction (Figure 6G). Supplementary patient‐level comparisons showed no FDR‐significant difference in pTST ratio or TCR repertoire metrics, whereas the TST score and Pai Tpex score differed after FDR correction and were higher in non‐MPR patients (Figure S6C). Receiver operating characteristic analysis showed that the MPR score achieved AUC = 0.753, compared with AUC = 0.611 for the Tpex ratio and AUC = 0.590 for Liu's Tpex signature score (Figure 6H and Table S3). To assess whether this association reflected a composite five‐program signal rather than a single gene program, we compared the fixed full MPR score with each individual component GP using the same patient‐level MPR endpoint and repeated stratified 5‐fold resampling (Figure S7A and Table S4). The full MPR score showed stable performance (full‐cohort AUC = 0.753; repeated 5‐fold mean AUC = 0.753 ± 0.005) and exceeded each single GP in resampling analyses (single‐GP mean AUC range, 0.561–0.730). These comparisons indicate that the MPR score captures patient‐level response‐state information distinct from pTST abundance, repertoire diversity, and selected Tpex‐signature baselines in this cohort, and that its association is not dominated by any single GP.

Consistent with these observations, a score‐only logistic model showed an association between patient‐level mean MPR score and MPR status (OR = 3.00 per one standard deviation increase; 95% CI 2.08–4.33; *p* = 4.1 × 10^−^^9^) (Figure 6I). Using the clinical covariates available from the original Liu et al. report, one‐covariate adjustment models preserved the MPR‐score association after adjustment for age, gender, cancer type, and stage group (Figure 6I and Figure S7B and Table S5). In patients with complete follow‐up data (*n* = 152; 30 recurrence events), higher MPR score was associated with improved recurrence‐free survival (RFS) in a univariate Cox model (HR per one standard deviation = 0.611; 95% CI 0.448‐0.833; = 0.00186), with similar directionality in stratified Cox sensitivity analyses where event counts were sufficient (Figure 6J and Figure S7C).

Kaplan‐Meier survival curves stratified by the median MPR score further showed longer RFS in the high‐score group (log‐rank *p* = 0.00664). Alternative metrics, including the Tpex ratio and previously established Tpex‐based signatures, showed weaker or non‐significant prognostic value (Figure 6K,L). Subgroup analyses further showed directionally retained MPR‐score associations across evaluable age, smoking, histology, stage, and sparse PD‐L1 TPS strata (Figure S7D), and one‐covariate RFS Cox sensitivity analyses showed consistent directionality where event counts were sufficient (Figure S7E). Across the available clinical annotations, covariate‐adjusted and subgroup analyses consistently retained the MPR‐score association, with sparse or unavailable fields handled according to predefined evaluability criteria. Together, these data support the MPR score as a retrospective patient‐level response‐state measure associated with pathological response and recurrence‐free survival in this cohort.

## 3 Discussion

The identification and functional characterization of tumor‐specific T cells (TSTs) are important for deciphering the mechanisms of immune checkpoint blockade (ICB) and guiding next‐generation immunotherapies. In this study, we present TSTScope, a multimodal deep learning framework that moves beyond simple data concatenation to create a biologically constrained, unified representation of T‐cell identity. By integrating transcriptional programs with TCR sequence features, our work provides an interpretable strategy for studying receptor‐linked T‐cell states associated with ICB response.

First, joint modeling guided by biological priors can help refine antigen‐specific signals from noisy scTCR‐seq data. A major challenge in current TCR analysis is that raw embeddings are often confounded by conserved germline V/J gene usage, which masks the critical antigen‐specificity signals driven by the CDR3 region [43, 44]. TSTScope addresses this by aligning TCR embeddings with gene‐program‐constrained transcriptional representations. In the evaluated benchmarks, this strategy reduced germline‐associated clustering and produced representations that aligned more closely with antigen specificity than single‐modality baselines and component‐removal controls. These results support multimodal integration as a useful approach for linking clonotype to function in the TME.

Second, applying TSTScope to ICB‐treated NSCLC cohorts indicates that the functional potential of pTSTs, rather than their abundance alone, is associated with therapeutic response. While previous strategies have focused on quantifying tumor‐infiltrating lymphocytes or broadly reactive clones [10, 45, 46], our analysis showed that the proportion of potential TSTs (pTSTs) in tumor tissue does not correlate with MPR. Instead, we identified a distinct functional dichotomy. pTSTs in non‐MPR patients were characterized by terminal exhaustion and senescence programs. Conversely, pTSTs in MPR patients exhibited a progenitor‐like state enriched for early activation, TCR signaling, and stemness modules [33, 35, 47]. This specific functional phenotype captured by our defined “MPR score” is consistent with the idea that clinical benefit from ICB depends in part on TSTs that retain plasticity to expand and differentiate, rather than only on the accumulation of terminally differentiated effectors.

These biological insights translate into the MPR score, a retrospective patient‐level response‐state measure. In an independent Liu cohort, the MPR score was associated with pathological response and recurrence‐free survival, and it showed favorable point estimates relative to several Tpex‐ and repertoire‐based metrics. External state signatures captured related biology in some patient‐level comparisons, but the MPR score organizes this signal around a receptor‐linked functional niche defined by tumor‐reactivity and proliferative potential. These findings support the utility of TSTScope for discovery and cohort‐level response‐state analysis, and they motivate prospective biomarker studies.

Several extensions will broaden the scope of TSTScope. Although the current framework focuses on CD8^+^ T cells, incorporating CD4^+^ helper programs would expand coverage of anti‐tumor immunity. The use of predefined gene programs supports interpretability and robustness, but it may limit the discovery of novel, uncharacterized biological pathways. Furthermore, high‐quality paired scRNA‐seq and scTCR‐seq data remain a primary input for linking receptor specificity with functional state, and validation across additional cancer types will be important for defining the generality of the MPR score. As highlighted in single‐cell immune repertoire analysis frameworks [48], scTCR‐seq interpretation requires explicit handling of receptor‐chain calls and contig quality. Consistent with repertoire‐analysis practice that prioritizes productive receptor calls with the strongest molecular support, the current TCR branch uses beta‐chain CDR3 embeddings and retains the highest‐UMI productive beta‐chain contig when multiple beta‐chain contigs are assigned to the same cell. Nevertheless, this strategy does not fully model paired alpha/beta receptor biology and may discard secondary beta‐chain information from true dual‐chain cells or technical multiplets. Paired alpha/beta‐aware modeling and explicit dual‐chain receptor representation therefore remain important extensions. Spatially resolved receptor‐state mapping is another important direction. Spatial profiling can resolve tumor, stromal, and neighborhood context, and future datasets combining spatial resolution, paired receptor information, and clinical outcomes will enable direct mapping of receptor‐defined functional states in tissue.

In conclusion, TSTScope provides an interpretable map of T‐cell heterogeneity that links antigen specificity with functional state. By shifting attention from the quantity to the functional quality of tumor‐specific T cells, it nominates a response‐associated phenotype that helps explain variation in ICB response. These insights provide a basis for testing whether preserving or expanding this “MPR‐positive” T‐cell state can improve clinical outcomes.

## 4 Materials and Methods

### 4.1 TSTScope Model Design

To jointly analyze single‐cell gene expression and TCR sequence data while preserving biological interpretability, we developed TSTScope, a multi‐modal Conditional Variational Autoencoder (CVAE) framework. TSTScope aligns scRNA‐seq profiles and TCR embeddings into a shared, biologically meaningful latent space. The model comprises two parallel encoding branches, one for gene expression and another for TCR embeddings, projecting data into a shared latent space constrained by prior biological knowledge in the form of predefined T cell functional GPs (Figure 1).

### 4.2 Gene Expression Module

For the transcriptomic modality, we implemented a CVAE architecture [22] incorporating explainable constraints within the decoder to capture biologically interpretable latent representations. Let $x_{g}\in R^{G}$ denote the gene expression values for a cell, where G corresponds to the subset of genes annotated in at least one prior‐defined GP. The encoder *E_g_* compresses the high‐dimensional input *x_g_* into a low‐dimensional latent space by estimating a mean vector and a variance vector σ_*g*_. A probabilistic latent vector $z_{g}\in R^{L}$ is generated via reparameterization, where the *L* corresponds to the number of GPs derived from prior knowledge.

To enforce interpretability, the decoder *D_g_* utilizes a structured constraint mechanism governed by a GP matrix $M\;\in \;{\left\{0,1\right\}}^{L\times G}$, a binary mask where the entry *M_ij_* =  1 indicates that gene *j* is annotated to Gene Program *i*, and 0 otherwise. The GP matrix was constructed from 50 prior‐defined T‐cell functional programs, with gene members, literature source, functional category, gene‐set size, and downstream score usage documented in Table S1; these GP definitions were not derived from the Caushi or Liu evaluation cohorts. The *W_dec_*, are constrained via element‐wise multiplication: *W_constrained_* = *W_dec_* ⊙*M*. This ensures that a latent GP variable contributes to a gene's reconstruction only if biologically associated. The predicted mean expression μ_*rec*_ is obtained by scaling the relative abundance with the total library size *s* of each cell. The reconstruction is modeled using a Negative Binomial (NB) distribution as shown in Equation (1).

(1) $$p\;\left(x_{g}|z_{g},s,b\right)=NB\left(\;x_{g};\mu_{rec},\theta \right)$$

where μ_*rec*_ =  *s* · softmax(*W_constrained_* ^*T*^ *z_g_*) represents the predicted mean expression adjusted by the library size *s*, and θ represents the gene‐specific dispersion parameters conditioned on batch *b*. The reconstruction loss is defined as the negative log‐likelihood of the gene expression counts, as represented in Equation (2):

(2) $$L_{gene}=-\sum_{i=1}^{N}\sum_{j=1}^{G}logNB\left(x_{ij};\mu_{ij},\theta_{j}\right)$$

### 4.3 TCR‐Branch Module

We utilized the fine‐tuned TCR‐BERT model [21] in inference mode to extract initial TCR embeddings denoted as $x_{tcr}\in R^{D}$. The TCR encoder, *E_tcr_* is designed with an architecture analogous to the gene expression encoder. It accepts the initial embeddings *x_tcr_* as input and projects them into a probabilistic latent space by estimating the Gaussian distribution parameters: a mean vector μ_*tcr*_ and a log‐variance vector σ_*tcr*_. Crucially, the dimensionality of this latent space is set to *L*, ensuring strictly consistent dimensionality with the gene expression latent space. A latent vector $z_{tcr}\in R^{L}$ is subsequently obtained via reparameterization sampling. A symmetric Multi‐Layer Perceptron (MLP) decoder, *D_tcr_* reconstructs the original TCR embeddings, with loss defined as Mean Squared Error (MSE) between the input embeddings and the reconstructed output, as shown in Equation (3):

(3) $$L_{tcr}={\left|\left|x_{tcr}-D_{tcr}\left(z_{tcr}\right)\right|\right|}_{2}^{2}\;$$

### 4.4 Joint Latent Space Alignment and Fusion

To integrate the two modalities, we implemented a clonotype‐guided alignment strategy that synchronizes the latent representations of the two modalities while preserving biological distinctiveness. We define a Center‐Mean alignment loss *L_align_* that minimizes the distance between the centroids of cells sharing the same TCR clonotype across modalities. Let *C* be the set of unique TCR clonotypes present in the current mini‐batch. For each specific clonotype c∈C, we compute the prototype (mean) latent vectors for both modalities as shown in Equation (4):

(4) $${\bar{\mu }}_{g}^{\left(c\right)}=\frac{1}{N_{c}}\;\sum_{i\in I_{c}}\mu_{g}^{\left(i\right)},\;{\bar{\mu }}_{tcr}^{\left(c\right)}=\frac{1}{N_{c}}\;\sum_{i\in I_{c}}\mu_{tcr}^{\left(i\right)}$$

where $I_{c}$ denotes the set of indices for cells belonging to clonotype *c*, and *N_c_* is the number of such cells.

The alignment loss consists of three components, including inter‐modality consistency by applying both Mean Squared Error (MSE) and Cosine Similarity loss between the paired Gene (${\bar{\mu }}_{g}^{\left(c\right)}$) and TCR (${\bar{\mu }}_{tcr}^{\left(c\right)}$) prototypes, and intra‐modality distinctiveness by applying a contrastive loss specifically to the gene prototypes to encourage distinctiveness among different prototype representations. The total alignment loss is defined as in Equations (5) and (6):

(5)

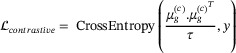

(6) $$\begin{array}{ccc} L_{align} & = & \lambda_{1}\;\mathrm{MSE}\left(\mu_{g}^{\left(c\right)},\mu_{tcr}^{\left(c\right)}\right)+\lambda_{2}\left(1-\mathrm{CosSim}\left(\mu_{g}^{\left(c\right)},\mu_{tcr}^{\left(c\right)}\right)\right)\\ & & +\;\lambda_{3}L_{contrastive} \end{array}$$

where τ is a temperature parameter and *y* represents the identity labels for the clonotypes. Weights used in alignment loss were set to λ_1_ = 1, λ_2_ = 10, and λ_3_ = 1. This objective pulls cells with identical TCRs toward the same functional state while ensuring that different clonotype prototypes remain distinguishable in the gene space.

Finally, a fused latent variable $z_{fusion}\in R^{L}$ is computed via a linear combination of latent representation *z_g_* and *z_tcr_*, controlled by a fixed weighting hyperparameter λ  =  0.5, followed by Layer Normalization.

### 4.5 Joint Latent Space and Loss Function

The model is trained end‐to‐end by minimizing a composite loss function as in the Equation (7):

(7) $$\begin{array}{ccc} L_{total} & = & \lambda_{rec\_gene}L_{gene}+\lambda_{rec\_tcr}L_{tcr}\\ & & +\;\lambda_{KL}D_{KL}\left(q\left(z|x\right)∥p\left(z\right)\right)+\lambda_{align}L_{align} \end{array}$$

where $L_{NB}$ is the negative log‐likelihood of the gene reconstruction, *D_KL_* is the Kullback‐Leibler divergence regularizing the latent distributions toward a standard normal prior *N*(0, *I*), and $L_{align}$ is the joint alignment loss. The weighting coefficients λ balance the reconstruction accuracy, latent space regularization, and multi‐modal alignment. We used the Adam optimizer for model training. The hyperparameters used in this model are listed in Table S6.

### 4.6 Datasets and Preprocessing

#### 4.6.1 10x Dataset

A publicly available dataset was obtained from the 10x Genomics website, comprising paired scRNA‐seq and scTCR‐seq profiles from four healthy donors, with TCR antigen specificities experimentally validated using peptide‐MHC (pMHC) tetramers [49]. From 33 804 CD8^+^ T cells from donor 2 annotated with TCR specificities spanning 29 antigen classes, the 6 antigen specificities with the largest clonal expansions were selected for antigen specificity classification, enabling evaluation of TSTScope's capacity to capture antigen specific TCR features.

#### 4.6.2 Caushi et al. Cohort

This dataset includes paired scRNA‐seq and scTCR‐seq profiles from 16 anti‐PD‐1‐treated patients with known MPR outcomes [28]. After quality filtering, the dataset comprised 188 804 CD8^+^ T cells across 88 samples. The MANA/Viral antigen‐labeled validation object contained 3503 cells from 7 patients, including 2083 neoantigen‐specific T cells (MANA) and 1420 viral‐specific T cells. The paired‐chain antigen‐labeled subset contained 2722 cells from 6 patients.

#### 4.6.3 Chen et al. Spatial NSCLC Immunotherapy Resource

The processed GeoMx table contained Q3‐normalized CTA expression from 5 NSCLC cases and 285 spatial ROIs across 5 mapped case strata. These ROIs included 86 stem‐immunity ROIs, 78 TLS‐like ROIs, 74 immunity‐hub ROIs, 40 non‐hub ROIs, and 7 excluded ROIs [39]. The CTA panel covered 137 matched program‐gene entries, corresponding to 128 unique genes across the component gene programs of the independently defined TSTScope MPR score. This resource enabled an orthogonal spatial association analysis between the independently defined MPR score and response‐associated stem‐immunity hub location.

#### 4.6.4 Liu et al. Cohort

This cohort comprises a comprehensive atlas of 1 254 749 tumor‐infiltrating immune cells with paired scRNA‐seq and scTCR‐seq data from anti‐PD‐1 treated NSCLC patients [40]. Based on the original cell‐type annotations provided by Liu et al., 219 842 intratumoral CD8^+^ T cells were initially selected without additional quality‐control filtering. Patients with fewer than 100 intratumoral CD8^+^ T cells were excluded. After filtering, 219 188 CD8^+^ T cells from 221 patients were retained for downstream analyses using the full TSTScope pipeline. Among these patients, 220 had available MPR status, and 152 had recurrence‐free survival data.

#### 4.6.5 scRNA‐seq Preprocessing

scRNA‐seq data generated using the 10x Genomics platform and preprocessed with Cell Ranger were analyzed using Scanpy (v1.8.1). Cells with fewer than 200 detected genes or more than 15% mitochondrial gene expression were excluded, and genes detected in fewer than 3 cells were removed. Quality‐controlled expression counts were directly used as input for TSTScope, scAtlasVAE and ExpiMap. For PCA‐based analyses, expression counts were further normalized to 10 000 transcripts per cell and log‐transformed. Highly variable genes were identified by selecting the top 4000 genes, with T cell receptor‐related genes excluded based on IMGT annotation [50] resulting in 3,894 genes retained for downstream analyses. Dataset‐specific batch or sample identifiers were handled using “sample_name” for the Caushi cohort, “sample” for the Liu cohort, and “batch” for the 10x donor benchmark. Biological annotations, including antigen label, and MPR status, were used for evaluation, visualization, and sensitivity analyses, not as response labels for model training or held‐out antigen‐label classification.

#### 4.6.6 scTCR‐seq Preprocessing

scTCR‐seq data were obtained as the Cell Ranger processed contig annotation files. scRNA‐seq and scTCR‐seq data were integrated using shared cellular barcodes. The current TSTScope production workflow uses TCR β‐chain CDR3 amino acid sequences as the receptor input. When multiple productive beta‐chain contigs were assigned to the same cell, the productive beta chain with the highest UMI count was retained as the representative beta‐chain sequence. Cells without an available productive TCR β‐chain CDR3 amino acid sequence were excluded from analyses requiring TCR input.

### 4.7 Method Comparisons and Implementation Details

We systematically benchmarked TSTScope against 6 representative single‐modality feature embedding methods covering both TCR sequence and transcriptomic analysis, and included 2 paired‐modality scRNA‐seq/scTCR‐seq integration controls for paired‐chain benchmark analyses.

#### 4.7.1 TCR Sequence Embedding Models

We evaluated 3 baselines for TCR representation learning:

**scCVC** [7]: We performed model inference using the scCVC foundation model. The TCR β amino acid sequences were extracted from the single‐cell dataset and processed through an Embedding Wrapper utilizing a pre‐trained Transformer checkpoint. For each sequence, we extracted features from the final hidden layer and applied a mean‐pooling strategy to aggregate token‐level information into a fixed‐size embedding vector.
**TCR‐BERT & Fine‐tuned TCR‐BERT** [21]: The TRB sequences were extracted from the single‐cell metadata and **processed** through the TCR‐BERT encoder with a pre‐trained baseline version and a fine‐tuned version optimized for specific downstream tasks. To capture deep contextual representations of the amino acid motifs, features were retrieved from the final hidden layer using a layer‐wise extraction strategy. To condense the sequence‐length outputs into cell‐level representations, a mean‐pooling method was employed, averaging the hidden states across all tokens to produce a fixed‐dimensional latent vector for each cell.

#### 4.7.2 Transcriptomic Feature Extraction Methods

For gene expression analysis, we compared TSTScope against 3 established workflows:

**ExpiMap** [22]: We applied ExpiMap as a transcriptomic GP‐constrained variational baseline using the same predefined GP gene sets used by TSTScope (Table S1). The resulting latent embeddings provide a biologically regularized RNA‐only representation in which each dimension corresponds to a specific functional program.
**scAtlasVAE** [30]: Utilizing a reference‐mapping paradigm, transcriptomic profiles were projected into a high‐dimensional latent space defined by a pre‐trained reference model. We employed the human CD8^+^ T cell atlas as the structural scaffold for embedding inference. By aligning query cells to this established reference, we extracted fixed‐size latent vectors that capture the relative positioning of individual cells within the broader immunological landscape.
**PCA with Harmony** [29]: Representing the scRNA‐seq data integration baseline, we utilized identified HVGs to focus on the most informative transcriptomic features. Following Principal Component Analysis (PCA) to reduce dimensionality, we applied the Harmony **algorithm** to perform iterative batch correction. This strategy aligns cells across disparate donors and experimental conditions, yielding a corrected principal component space used as the final embedding representation.

#### 4.7.3 Paired‐Modality scRNA‐seq/scTCR‐seq Integration Methods

For paired‐chain benchmark analyses, we compared TSTScope against 2 representative multimodal integration workflows:

**mvTCR** [3]: mvTCR is a paired scRNA‐seq/scTCR‐seq integration framework that uses a mixture‐of‐experts (MoE) architecture to **combine** modality‐specific information into a shared latent space. The model encodes transcriptomic profiles and TCR sequence features through modality‐aware components and infers a multimodal representation for each cell. In our implementation, gene‐expression profiles and paired alpha/beta‐chain TCR information were provided as model inputs, and the inferred joint latent representation was extracted as the mvTCR embedding.
**UniTCR** [5]: UniTCR is a contrastive cross‐modal representation‐learning framework that aligns TCR sequence and transcriptomic information through paired profile‐to‐TCR and within‐modality objectives. The model separately encodes TCR and gene‐expression inputs and **projects** them into normalized latent representations for cross‐modal comparison. In our implementation, UniTCR was adapted to the available beta‐chain CDR3 amino acid sequence and matched transcriptomic profile. Because the paired‐cell corpus was large, we implemented the paired‐distance objective in mini‐batches rather than precomputing a full cell‐by‐cell distance matrix. During training, pairwise targets were computed within each mini‐batch, using cosine distance between standardized gene‐expression profiles as the profile‐pair distance and cosine similarity between Atchley‐factor‐encoded beta‐chain CDR3 vectors as the TCR‐pair similarity. The normalized profile and TCR branch outputs were concatenated as the UniTCR joint embedding.

### 4.8 Component Ablation and Detailed Model Evaluation

#### 4.8.1 Antigen‐Specificity Embedding Classifier

Antigen‐specificity classification in the 10x donor benchmark was performed after selecting one representative cell per unique TCR clonotype label and restricting the analysis to the 6 most frequent peptide‐antigen classes. Fixed TCR embeddings from TSTScope, scCVC, TCR‐BERT, and fine‐tuned TCR‐BERT were evaluated under identical stratified 5‐fold cross‐validation splits with shuffling and random_state of 42. In each fold, embeddings were used as input features to an RBF‐kernel support‐vector classifier with probability = True. Performance summaries were computed across folds using accuracy, weighted precision, weighted recall, weighted F1 score, and weighted multiclass ROC‐AUC with a one‐vs‐one criterion. For the ROC visualization, the highest‐AUC fold for each embedding was used to plot micro‐average, macro‐average, and per‐antigen one‐vs‐rest ROC curves from held‐out class‐probability estimates.

#### 4.8.2 Embedding‐Based Antigen‐Label Benchmark Classifiers

Fixed latent embeddings from each evaluated method were standardized using training‐fold statistics and evaluated by Euclidean k‐nearest‐neighbor (kNN) label transfer with k = 30. For each leave‐one‐patient‐out (LOPO) fold, the held‐out patient's cells were excluded from feature standardization and any training‐fold fitting, and pooled predictions were evaluated only after applying the fitted transform to the held‐out patient.

#### 4.8.3 Component‐Removal Controls

The full TSTScope reference used the production joint embedding from the full Caushi CD8^+^ training cohort and was evaluated on the 3503 MANA/Viral antigen‐labeled cells. Component‐removal variants were retrained after removing one model constraint at a time: transcriptomic reconstruction, clonotype alignment, or the gene‐program mask. All variants were evaluated under the same LOPO kNN antigen‐label recovery protocol. This analysis was used to test representation‐learning sensitivity under a matched recovery protocol, while retaining the provenance distinction between the production reference and component‐removal retraining controls.

#### 4.8.4 Paired‐Chain Benchmark Controls

The analysis was restricted to the 2722 MANA/Viral antigen‐labeled cells with paired alpha/beta‐chain information. The TSTScope comparison used the subsetted TSTScope joint embedding. For a fair transcriptomic‐input comparison, mvTCR and UniTCR used the same gene feature set as TSTScope. mvTCR MoE was trained with paired‐chain information on 143 698 paired‐chain CD8^+^ cells and evaluated on the same paired‐chain antigen‐labeled subset. UniTCR was run as a single‐beta‐chain adapted joint control on the same paired‐chain evaluation subset. MANA/Viral labels were used only for held‐out LOPO evaluation, not for model training.

#### 4.8.5 Latent‐Space Mixing and Label‐Preservation Evaluation

To evaluate patient, tissue, and biological‐label structure in learned spaces, we visualized UMAP distributions and quantified nearest‐neighbor same‐label preservation, Local Inverse Simpson's Index (LISI), across embeddings. Higher patient LISI was interpreted as stronger patient or batch mixing, whereas lower MPR‐label LISI and higher same‐label neighbor fraction indicated stronger local preservation of response‐state labels. Together, these analyses characterized how each latent space balanced cohort‐level mixing with preservation of pTST‐ or MPR‐related local structure.

### 4.9 Gene Program Activity Quantification and Differential Analysis

To ensure the biological interpretability of the latent space, the sign of each gene program (GP) latent variable was aligned with its corresponding gene expression profile. For each GP, an aggregate expression score was calculated based on its constituent gene set. We then determined the Pearson correlation between this expression score and the corresponding latent dimension. To ensure that positive latent values consistently represent higher biological activity, latent values were multiplied by a direction factor derived from the sign of this correlation. These direction‐corrected GP activity scores were then standardized across all cells using Z‐score normalization to facilitate downstream visualization and comparative analyses.

Differential GP activity between cell populations was assessed using a non‐parametric Bayesian framework. For each comparison, empirical distributions of GP activity were generated via bootstrapping (1,000 iterations per group). We estimated the posterior probability that activity in the target group (A) exceeded that in the reference group (B). The significance of this difference was quantified using the Bayes factor (BF), calculated as in Equation (8):

(8) $$K=\;\ln\frac{p\left(A>B\right)}{1-p\left(A>B\right)}$$

GPs were identified as significantly differentially active if the absolute Bayes factor exceeded a threshold of 1.1.

### 4.10 Prediction and Evaluation of Tumor‐Specific T Cell Subpopulations

To quantify tumor specificity, we defined a TST score as the net difference between the aggregate expression of positive Gene Program (GP) markers (TUMOR_SPECIFIC, TERM_TEX, OXPHOS_TEX) and negative markers (EARLY_TEM, NAÏVE, TCR_SIGNALING).

The distribution of TST scores was modelled with a two‐component Gaussian Mixture Model (GMM). To ensure a robust fit, we employed multiple random initializations and selected the final model based on the Bayesian Information Criterion (BIC). We assessed component separability by calculating the ratio of the distance between means to the sum of their standard deviations. The classification threshold was determined by the intersection of the two weighted Gaussian densities via numerical root‐finding. In cases where an intersection was mathematically invalid or separability fell below a predefined heuristic, the global median score was used as the fallback threshold. Cells exceeding this threshold were labeled as potential TSTs (pTSTs), while the remainder were classified as non‐pTSTs. The fixed TST score was evaluated in the Caushi antigen‐labeled dataset after restricting to MANA‐ or Viral‐labeled cells, yielding 3503 labeled cells from 7 patients for the LOPO procedure. In each fold, score orientation and threshold fitting were performed using training patients only and then applied unchanged to the held‐out patient. Performance was summarized using pooled held‐out AUC and average precision with patient‐level bootstrap uncertainty.

We further quantified and visualized clonotype composition and functional gene set enrichment profiles to characterize differences between pTST and non‐TST groups. Clonal analysis was restricted to expanded clonotypes (size > 5), with log‐transformed proportions calculated for group‐wise comparisons. Functional module scores, including TRM, Checkpoint, Cytotoxicity, and TCR signaling signatures, were computed using the “score_genes” function in Scanpy. Statistical significance of enrichment differences was assessed using the Mann‐Whitney U test. Differential gene expression (DGE) analysis was performed for both pTST vs. non‐TST and MANA vs. Viral contrasts using the Wilcoxon rank‐sum test with the “rank_genes_groups” function in Scanpy. To evaluate the consistency of transcriptional programs, concordance of gene rankings across comparisons was quantified using Pearson correlation coefficients with associated p‐values and visualized via scatter plots.

### 4.11 Benchmarking Against Tumor‐Reactive Signatures

To evaluate the predictive performance of TSTScope in identifying pTSTs, we compared the TST score against several established transcriptomic signatures validated for tumor‐reactive CD8^+^ T cells. These benchmarks represent diverse approaches to defining tumor specificity, ranging from single‐gene markers to large‐scale transcriptomic programs.

#### 4.11.1 MANA Score

Calculated following the methodology of Zeng et al., this score utilizes a weighted expression of CXCL13, ENTPD1, and IL7R to differentiate neoantigen‐specific (MANA) CD8^+^ T cells from bystander populations [14].

#### 4.11.2 CXCL13 Score

Evaluated as a high‐resolution single‐gene proxy for tumor reactivity by quantifying *CXCL13* expression at the single‐cell level [10].

#### 4.11.3 NeoTCR8 Score

A comprehensive 243‐gene signature defined by Lowery et al., designed to capture the transcriptional landscape of neoantigen‐reactive T cells derived from metastatic cancer cohorts, the signature scores were computed on raw expression matrices using gene set based scoring and used for downstream evaluation [12].

#### 4.11.4 Oliveira Score

Computed using the gene set defined by Oliveira et al., representing transcripts significantly upregulated in tumor‐specific CD8^+^ tumor‐infiltrating lymphocytes (TILs) relative to virus‐specific bystander T cells [11]. The signature scores were computed on raw expression matrices using gene set based scoring and used for downstream evaluation.

### 4.12 Formulation and Evaluation of the MPR Score

To quantify the therapeutic potential of the tumor‐reactive T cell repertoire, we developed the Major Pathological Response (MPR) Score, a composite transcriptomic metric. The development and validation of this score proceeded through feature identification, score formulation, sensitivity analysis, and comparative benchmarking.

#### 4.12.1 Identification of MPR‐Associated Gene Programs

To identify transcriptional programs associated with clinical response, single‐cell GP activities within intratumoral pTSTs were first aggregated to the patient level by calculating mean activity scores for each patient sample. We assessed differential GP activity between patients who achieved MPR and non‐MPR using a two‐sided Mann–Whitney U test. Gene programs demonstrating a statistically significant difference after Benjamini‐Hochberg FDR adjustment (*q* < 0.05) were selected as candidate features for the final score.

#### 4.12.2 Formulation of the MPR Score

The MPR score was designed to integrate multiple signals of effector potential into a single metric. At the single‐cell level, the score was defined as the summed activity of the five response‐associated GPs identified in the preceding step (IFN_RESPONSE, TCR_SIGNALING, TCM, NEOTCR_CD8, and MODULE_1). Patient‐level MPR scores were then calculated by averaging single‐cell MPR scores across each patient's intratumoral pTST population. This approach allows for a robust representation of the functional state of the T cell repertoire rather than relying on individual gene expression.

#### 4.12.3 MPR‐Score Sensitivity Analysis

To evaluate whether the current MPR‐score association was dominated by a single gene program, we performed a fixed‐formula sensitivity analysis in the Liu dataset. The five‐term MPR formula (IFN_RESPONSE, TCR_SIGNALING, TCM, NEOTCR_CD8, and MODULE_1) was compared with each individual component GP using the same patient‐level MPR endpoint and repeated stratified 5‐fold patient‐level resampling without training a classifier or re‐estimating score weights. Sensitivity was summarized using full‐cohort patient‐level AUC and repeated 5‐fold mean AUC (Table S4).

#### 4.12.4 Spatial Stem‐Immunity Hub Analysis

The Chen et al. spatial resource was used to evaluate whether spatial tumor‐context readouts aligned with the TSTScope MPR score. For ROI‐level GeoMx CTA analysis, Q3‐normalized expression values were log‐transformed and Z‐score standardized within each case for each gene. The independently defined MPR score was projected into each ROI by scoring the matched CTA genes for IFN_RESPONSE, TCR_SIGNALING, TCM, NEOTCR_CD8, and MODULE_1 and averaging the five program scores. ROI location differences were evaluated using Kruskal‐Wallis tests, FDR‐adjusted pairwise Mann‐Whitney U tests, and within‐case location contrasts. Case‐adjusted logistic regression was used to test whether higher standardized values of the independently defined MPR score were associated with stem‐immunity hub ROI location after accounting for case.

#### 4.12.5 Benchmarking Against Established Biomarkers

To evaluate predictive performance, we benchmarked the MPR score against established transcriptomic signatures, cell‐state abundance metrics, and repertoire‐based metrics associated with tumor‐reactive or progenitor exhausted (Tpex) CD8^+^ T cells.

#### 4.12.6 Liu et al. Tpex ratio

The patient‐level Tpex ratio was calculated from the Liu cell‐state annotations as the number of Tpex‐labeled CD8^+^ T cells divided by the combined number of Tpex‐labeled and terminal exhausted CD8T_terminal_Tex_LAYN cells. This ratio was used as a cell‐state abundance benchmark in MPR and RFS analyses [40].

#### 4.12.7 Liu et al. Tpex Signature

Gene list derived from longitudinal single‐cell profiling of NSCLC patients undergoing anti‐PD‐1 therapy, capturing a precursor exhausted T cell state characterized by stem‐like and checkpoint‐associated markers [36]. The signature scores were computed on raw expression matrices using gene set based scoring and used for downstream evaluation.

#### 4.12.8 Gueguen et al. Tpex Signature

Gene list describing a memory‐like CD8^+^ T cell population with regenerative capacity identified in NSCLC [37]. The signature scores were computed on raw expression matrices using gene set based scoring and used for downstream evaluation.

#### 4.12.9 Pai et al. Tpex Signature

Gene list defined through multi‐regional lineage tracing of tumor‐specific T cells [38], marking a clonally expandable progenitor population persisting across lymphoid and tumor compartments. The signature scores were computed on raw expression matrices using gene set based scoring and used for downstream evaluation.

#### 4.12.10 TCR Repertoire Shannon entropy

Calculated from the frequency distribution of unique TCR clonotypes within each patient [41], as defined in Equation (9):

(9) $$H=-\sum_{i}^{p_{i}}\log\left(p_{i}\right)$$

where *p_i_* denotes the relative abundance of clonotype *i*.

#### 4.12.11 TCR Repertoire Clonality

Defined as the complement of normalized Shannon entropy [41], as defined in Equation (10):

(10) $$\mathrm{Clonality}=1-\frac{H}{\log\left(N\right)}\;$$

where *N* is the total number of unique clonotypes, yielding values ranging from 0 (fully polyclonal) to 1 (highly oligoclonal).

### 4.13 Performance Evaluation of Patient ICB Response Clinical Outcomes

The MPR score in the context of ICB therapy was assessed through a multi‐level statistical framework designed to evaluate patient‐level discriminative association, covariate‐aware support, and longitudinal survival association.

For the independent Liu dataset response analyses, pTST and MPR‐score summaries were aggregated at the patient level before ROC, logistic‐regression, and RFS analyses, so cells were not treated as independent clinical observations. Pathological response was represented as a binary patient‐level endpoint, with pCR and MPR patients grouped as MPR and compared against non‐MPR patients. Available Liu clinical annotations supported one‐covariate adjustment for age, gender, cancer type, and stage group, and subgroup consistency checks across age, smoking status, histology, stage, and partial PD‐L1 TPS strata (Table S5). TMB and ECOG status were not available in the public tables.

#### 4.13.1 Discriminative Analysis

Differences in patient‐level MPR score distributions between MPR and non‐MPR patients were evaluated using two‐sided Mann‐Whitney U tests. The score's ability to differentiate clinical response was quantified using Receiver Operating Characteristic (ROC) curve analysis. Area Under the Curve (AUC) values were calculated from continuous patient‐level score distributions using scikit‐learn (v1.3.0), facilitating direct comparison of the MPR score against established molecular benchmarks, including Tpex signatures, Tpex ratio, and TCR repertoire metrics. Patient‐level bootstrap resampling was used to estimate AUC confidence intervals for these benchmark summaries.

#### 4.13.2 Logistic Regression and Covariate Adjustment

To assess whether the MPR‐score association was retained after accounting for available clinical covariates, we fitted score‐only and parsimonious one‐covariate logistic regression models using statsmodels (v0.14.0). In the score‐only model, binary MPR status was regressed on the patient‐level mean MPR score after Z‐score standardization, so the odds ratio represents the change in odds of MPR per one standard deviation increase in MPR score. One‐covariate models added one clinical covariate at a time to the standardized MPR score. These covariates included age, gender, cancer type, and stage group when model convergence and covariate support were adequate. Categorical covariates were dummy‐coded. Smoking history was excluded from the final adjusted summaries because the corresponding model did not fully converge. Partial PD‐L1 TPS was too sparse for logistic adjustment and was evaluated in subgroup analyses. TMB and ECOG status were unavailable. Results are reported as odds ratios (ORs) with 95% confidence intervals (CIs) and model‐based *p* values.

#### 4.13.3 Survival Analysis

RFS analyses were restricted to patients with complete patient‐level score, follow‐up time, and recurrence‐event annotations. Cox proportional hazards models were implemented in lifelines (v0.28.0). For univariate survival screening, continuous patient‐level scores were Z‐score standardized, and each score was modeled separately to estimate the hazard ratio per one standard deviation increase. The primary univariate analysis tested patient‐level mean MPR score in 152 patients with complete RFS annotations and 30 recurrence events. Alternative biomarkers, including TST score, pTST ratio, Tpex ratio, MANAscore, CXCL13 score, NeoTCR_CD8 score, TCR clonality, and established Tpex‐based scores, were analyzed under the same univariate framework.

For Kaplan‐Meier analysis, patients were divided into high‐ and low‐score groups using the median of MPR score, Tpex ratio, or Liu Tpex score, and survival curves were compared using the log‐rank test. Covariate‐aware RFS sensitivity was evaluated in two ways. First, one‐covariate Cox models added age, gender, smoking history, or cancer type individually to the standardized MPR score. Second, subgroup Cox analyses fitted the standardized MPR score within prespecified clinical strata, including age, smoking status, histology, stage, MPR status, anti‐PD‐1 agent, treatment‐cycle group, and PD‐L1 TPS group. Pre‐treatment stage adjustment and event‐limited subgroup estimates were excluded because of limited recurrence‐event support. Hazard ratios, 95% confidence intervals, model *p* values, and Harrell's Concordance Index (C‐index) were reported for Cox models.

### 4.14 Statistical Analysis

Statistical analyses were performed in Python 3.10.14, using functions in scikit‐learn (v1.3.0), statsmodels (v0.14.0), and lifelines (v0.28.0) packages. Continuous variables were compared using the two‐sided Mann‐Whitney U test. For DEG, DGP, and repeated Mann‐Whitney‐style group‐comparison families, *p* values were adjusted using the Benjamini‐Hochberg FDR procedure, and *q* < 0.05 was used for inferential claims. ROC, logistic‐regression, Cox, paired‐bootstrap comparison, and spatial ROI‐location analyses were summarized using their corresponding AUC confidence intervals, AUC differences, odds ratios, hazard ratios, Kruskal‐Wallis tests, FDR‐adjusted pairwise Mann‐Whitney U tests, or model *p* values as appropriate to each analysis family.

## Author Contributions

Conceptualization: Y.X.L., J.W.L., Methodology: J.W.L., S.W.C., Investigation: J.W.L., S.W.C., J.Y.C., Visualization: J.W.L., S.W.C., J.Y.C., F.A.W., C.X.Y., J.J.C., K.Y.W., Supervision: Y.X.L., J.W.L., L.L.L., Writing – original Draft: J.W.L., S.W.C., J.Y.C.

## Funding

This work was supported by National Key R&D Program 2023YFF1204701, Major Project of Guangzhou National Laboratory GZNL2025C01013, SRPG22007, National Natural Science Foundation of China 12371485, 82473482, Natural Science Foundation of Zhejiang Province Grants LY24H160007, Prevention and Control of Emerging and Major Infectious Diseases‐National Science and Technology Major Project 2025ZD01901900.

## Conflicts of Interest

The authors declare no conflicts of interest.

## Data and Materials Availability

The TCR antigen dataset is available from the 10x Genomics public repository (https://www.10xgenomics.com/datasets/cd‐8‐plus‐t‐cells‐of‐healthy‐donor‐2‐1‐standard‐3‐0‐2). The ICB response cohort datasets from Caushi et al. and Liu et al. are available through the Gene Expression Omnibus (GEO) under accession numbers GSE173351 and GSE243013, respectively. The Chen et al. spatial NSCLC immunotherapy resource is available through the Single Cell Portal under accession SCP2510 and through Zenodo under https://doi.org/10.5281/zenodo.11198494. The TSTScope implementation was developed using the scArches framework [31], and custom scripts used for data processing, model training, and analysis can be accessed on GitHub at https://github.com/YUZIXD/TSTScope. Processed data objects and integrated metadata required to replicate the findings of this study are available through Zenodo at https://doi.org/10.5281/zenodo.18162424.

## Supporting information




**Supporting File 1**: advs76983‐sup‐0001‐SuppMat.docx.

**Supporting File 2**: advs76983‐sup‐0002‐TableS1‐S6.zip.
