# Supplementary material for: TSTScope Unifies Single‐Cell Multi‐Omics to Identify Functional T Cell States Predictive of Immunotherapy Response
Source: Adv Sci (Weinh). 2026 Aug 5;13(60):e76983. doi: 10.1002/advs.76983 (PMC13440375; doi:10.1002/advs.76983)
Supplement: Supplementary file 1 [file ADVS-13-e76983-s002.docx]

Supplementary Materials for

**TSTScope Unifies Single-Cell Multi-Omics to Identify Functional T Cell States Predictive of Immunotherapy Response**

Shiwei Cao *et al.*

*Corresponding author. Email: li_yixue@gzlab.ac.cn;

[liu_junwei@gzlab.ac.cn](mailto:liu_junwei@gzlab.ac.cn);

liululu2001@zju.edu.cn.

**This PDF file includes:**

Figs. S1 to S7

Tables S1 to S6

**Fig. S1. Benchmarking of TCR embeddings and antigen-specificity distribution across T-cell subsets.** (**A**) Comparative performance of TSTScope and baseline TCR embeddings, including scCVC, TCR-BERT, and fine-tuned TCR-BERT, in the 10x donor antigen-specificity benchmark. One representative cell was selected per unique TCR clonotype label, and the analysis was restricted to the six most frequent peptide-antigen classes. Fixed TCR embeddings were evaluated using identical stratified 5-fold cross-validation splits and an RBF-kernel support-vector classifier. Data are presented as mean +/- s.d. across folds for accuracy, weighted precision, weighted recall, weighted F1 score, and weighted multiclass ROC-AUC. (**B**) Proportions of MANA-specific, Viral-1-specific, and Viral-2-specific T cells within annotated subtypes across the original Caushi annotation, scAtlasVAE, ExpiMap, and TSTScope. T-cell subtypes with MANA enrichment exceeding 85% are annotated with white numerals.

**Fig. S2. Distribution of benchmark tumor-reactive signatures across latent integration methods.** (**A-G**) UMAP visualizations of 188,804 CD8^+^ T cells from the Caushi NSCLC cohort (n = 16 patients) integrated using PCA+Harmony, scAtlasVAE, ExpiMap, and TSTScope. Cells are colored by normalized enrichment scores or expression levels for established tumor-reactive markers and signatures: (**A**) CXCL13 expression, (**B**) ENTPD1 expression, (**C**) NeoTCR-CD8 signature, (**D**) Oliveira-TST signature, (**E**) Tumor Reactive signature, (**F**) NeoTCRPBL signature, and (**G**) machine learning-derived MANA score. Color scales represent relative signature activity or gene expression within each latent representation.

**Fig. S3. Component-removal, paired-chain, and latent-space evaluation of TSTScope representations.** (**A**) UMAP visualizations of the three component-removal retraining controls after removing transcriptomic reconstruction, clonotype alignment, or the gene-program mask. The upper row shows all Caushi CD8^+^ cells, and the lower row shows the antigen-labeled MANA/Viral cells used for recovery evaluation. (**B**) Leave-one-patient-out (LOPO) k-nearest-neighbor antigen-label recovery on the 3,503 MANA/Viral antigen-labeled cells. Under the matched recovery protocol as in (A). AUC values and patient-level bootstrap confidence intervals are annotated in the panel. (**C**) UMAP visualizations of paired-chain benchmark embeddings for TSTScope, mvTCR mixture-of-experts (MoE), and the single-beta-chain adapted UniTCR control. The upper row shows paired-chain CD8^+^ cells, and the lower row shows the paired-chain MANA/Viral antigen-labeled cells used for recovery evaluation. (**D**) LOPO antigen-label recovery on the 2,722 paired-chain MANA/Viral antigen-labeled cells. Under the paired-chain benchmark protocol as in (C). AUC values and patient-level bootstrap confidence intervals are annotated in the panel. (**E**) Patient- and tissue-associated local structure across PCA+Harmony, scAtlasVAE, ExpiMap, and TSTScope embeddings, summarized by same-label nearest-neighbor fraction (Top) and inverse Simpson’s index (iLISI) (Bottom). (F) MPR-label and patient-label local structure across TSTScope and component-removal embeddings, summarized by same-label nearest-neighbor fraction (Top) and iLISI (Bottom).

**Fig. S4. Characterization of latent gene programs and evaluation of pTST identification.** (**A**) Rank-ordering of gene programs by absolute log Bayes factors within representative TST-associated subsets (GZMB^+^ Tex and HAVCR2^+^ Tex) and viral-associated subsets (IL7R^+^ Trm and GZMK^+^ Tem). (**B**) Dot plot showing mean gene-program activity and fraction of cells for MANA- and viral-associated gene programs across specific T-cell clusters. (**C**) Bivariate distribution of validated MANA-specific and viral-specific cells based on cumulative activity of positive and negative TST gene programs. (**D**) Comparison of Bayes factor scores across tumor-associated gene programs, including TUMOR_SPECIFIC, TUMOR_REACTIVITY, and NEOTCR_CD8. (**E**) UMAP and subset-level summaries showing enrichment of high TST-score pTSTs in subsets containing experimentally validated TSTs in the Caushi NSCLC cohort. (**F**) Proportions of predicted pTST and non-pTST labels and tissue-origin distribution of predicted pTSTs. (**G**) LOPO antigen-label precision-recall benchmark comparing the TST score with CXCL13 expression, MANAscore, NeoTCR_CD8 score, and Oliveira_TST score. Average precision (AP) values are annotated in the panel.

**Fig. S5. Relationship between the MPR score, Tpex-related signatures, and spatial stem-immunity hub organization.** (**A**) UMAP projections of intratumoral pTSTs from the Caushi NSCLC cohort (n = 56,885 cells), colored by enrichment scores for a canonical stemness signature and three independently defined Tpex gene signatures from Liu et al., Gueguen et al., and Pai et al. (**B**) Mean stemness and Tpex signature scores in intratumoral pTSTs from patients with MPR (n = 6) and non-MPR (n = 9). Statistical significance was assessed by two-sided Mann-Whitney U tests, with p values indicated. (**C**) Correlations between per-patient mean MPR scores and alternative stemness or Tpex signature scores across patients (n = 15). Pearson correlation coefficients and corresponding p values are indicated. (**D**) Box plots of the independently defined MPR score across Chen GeoMx ROI classes, including stem-immunity, TLS-like, immunity-hub, and non-hub regions. FDR-adjusted pairwise q values are annotated where shown. (**E**) Case-adjusted logistic ROI-location association models testing the association between standardized values of the independently defined MPR score and stem-immunity hub ROI location. Odds ratios, confidence intervals, and p values are annotated in the panel.

**Fig. S6. pTST inference and supplementary patient-level biomarker comparisons.** (**A**) TST-score distribution and two-component Gaussian mixture model used to infer pTSTs among 219,188 intratumoral CD8^+^ T cells from 221 Liu cohort patients, together with supporting summaries of the 85,400 inferred pTSTs across original CD8^+^ T-cell subsets. (**B**) Heatmap showing the overlap between original Liu CD8^+^ T-cell sub-cell types and TSTScope-defined pTST clusters. Values indicate the proportional contribution of each original sub-cell type to each pTST cluster. (**C**) Box plots comparing patient-level pTST ratio, TST score, TCR repertoire metrics, and Tpex-related scores between MPR and non-MPR patients. Benjamini-Hochberg FDR-adjusted q values across the displayed comparison family are annotated in each panel.

**Fig. S7. MPR-score sensitivity and clinical covariate support in the Liu cohort.** (**A**) Fixed-formula sensitivity panel comparing the five-term MPR score with each individual component gene program. Full-cohort AUC values and repeated stratified 5-fold patient-level AUC summaries are shown, with source metrics provided in Supplementary Table S4. (**B**) Forest plot of score-only and one-covariate logistic regression models for binary MPR status. Points show odds ratios for the standardized patient-level MPR score per one standard deviation increase, and horizontal bars show 95% confidence intervals after adding age, gender, cancer type, or stage group where evaluable. (**C**) Forest plot of stratified Cox sensitivity analyses for recurrence-free survival. Points show hazard ratios for the standardized MPR score per one standard deviation increase, horizontal bars show 95% confidence intervals, and n/e labels indicate the number of patients and recurrence events in each evaluable stratum. (**D**) Bar plot of patient-level MPR-score AUC for binary MPR status in the overall Liu cohort and across available clinical strata, including age, gender, smoking status, histology, stage, and partial PD-L1 TPS groups. Patient counts are annotated for each evaluable stratum. (**E**) Forest plot of one-covariate recurrence-free survival Cox sensitivity models. Points show hazard ratios for the standardized MPR score per one standard deviation increase, and horizontal bars show 95% confidence intervals after adding cancer type, age, smoking history, or gender

### Supplementary Tables

**Table S1. Curated T-cell gene programs used by TSTScope.** This table provides the 50 predefined T-cell gene programs used to constrain the TSTScope latent space, including gene-program index and name, signature type, reference or DOI, gene members, gene-set size, overlap with the analysis objects, downstream use in the TST or MPR score, and whether the gene program was derived from the Caushi or Liu evaluation cohorts. These gene-program definitions were curated independently of the Caushi and Liu evaluation cohorts.

**Table S2. LOPO TST-score antigen-label benchmark.** This table reports leave-one-patient-out antigen-label benchmark results for the fixed TST score and comparator transcriptomic scores in the Caushi MANA/Viral antigen-labeled cells. Metrics include AUC, average precision, patient-level bootstrap confidence intervals, bootstrap replicate counts, and resampling unit.

**Table S3. Patient-level MPR ROC benchmark.** This table reports patient-level ROC benchmark results for the MPR score, Tpex ratio, and Liu Tpex signature score in the Liu cohort. Metrics include AUC, average precision, patient-level bootstrap confidence intervals, bootstrap replicate counts, and resampling unit.

**Table S4. Five-term MPR-score sensitivity analysis.** This table reports the fixed full MPR score and each individual component gene program evaluated against the same patient-level MPR endpoint in the Liu cohort. Metrics include full-cohort patient-level AUC, repeated stratified 5-fold AUC summaries, fold-level AUC summaries, and deltas relative to the full five-term MPR score. No classifier training or score-weight re-estimation is used for this table.

**Table S5. Liu clinical covariate availability and evaluability.** This table provides patient-level Liu cohort clinical annotations matched to TSTScope patient-level scores, including age, gender, cancer type or histology, stage group, smoking status, partial PD-L1 TPS, treatment-related annotations, pathological response status, and recurrence-free survival fields where available. Missing fields are retained as NA, and the table documents which covariates supported one-covariate adjustment or subgroup analyses. TMB and ECOG status were not available in the public clinical tables.

**Table S6. TSTScope implementation, training settings, terminology, and benchmark protocols.** This table lists the final values and protocol settings for the TSTScope model and accompanying benchmark analyses, including architecture settings, implemented objective terms, loss weights, production and component-control training hyperparameters, antigen-specificity classifier settings, LOPO antigen-label recovery settings, component-removal controls, paired-chain mvTCR and adapted UniTCR controls, transcriptomic baselines, TST- and MPR-score analysis settings, and latent-space evaluation metrics.
